# Thermostability of a recombinant G protein-coupled receptor expressed at high level in mammalian cell culture

**DOI:** 10.1038/s41598-020-73813-7

**Published:** 2020-10-08

**Authors:** Alexei Yeliseev, Arjen van den Berg, Lioudmila Zoubak, Kirk Hines, Sam Stepnowski, Kyle Williston, Wanhua Yan, Klaus Gawrisch, Jonathan Zmuda

**Affiliations:** 1 National Institute on Alcoholism and Alcohol Abuse, NIH, Bethesda, MD 20892 USA; 2 ThermoFisher Scientific, 7335 Executive Way, Frederick, MD 21704 USA

**Keywords:** Biological techniques, Biophysics, Biotechnology, Drug discovery, Biochemistry, Biophysical chemistry, Proteins, Structural biology

## Abstract

Rational design of pharmaceutical drugs targeting integral membrane G protein-coupled receptors (GPCR) requires thorough understanding of ligand binding and mechanism of activation through high resolution structural studies of purified proteins. Due to inherent conformational flexibility of GPCR, stabilization of these proteins solubilized from cell membranes into detergents is a challenging task. Here, we take advantage of naturally occurring post-translational modifications for stabilization of purified GPCR in detergent micelles. The recombinant cannabinoid CB_2_ receptor was expressed at high yield in Expi293F mammalian cell cultures, solubilized and purified in Façade detergent. We report superior stability of the mammalian cell-expressed receptor compared to its *E.* *coli-*expressed counterpart, due to contributions from glycosylation of the N terminus and palmitoylation of the C terminus of CB_2_. Finally, we demonstrate that the mammalian Expi293F amino acid labelling kit is suitable for preparation of multi-milligram quantities of high quality, selectively stable isotope-labeled GPCR for studies by nuclear magnetic resonance.

## Introduction

Cannabinoid receptor CB_2_, a class A G protein-coupled receptor plays an important role in inflammation processes in various tissues including kidney, liver, and the gastrointestinal system. CB_2_ is an integral membrane protein primarily located in cells of immune and hematopoietic systems as well as in neuronal microglia^1^. Multi-milligram quantities of pure, stable, and homogenous receptor are required to study its structure and function by high resolution techniques. In addition, for characterization of the protein by nuclear magnetic resonance, it must be labeled with stable isotopes, either uniformly or at selected amino acid residues^2–4^.

We have previously described the expression of human CB_2_ receptor as a fusion with the maltose binding protein (MBP) in *Escherichia* *coli* cells^5–7^. The ability of *E.* *coli* to grow in media of defined composition facilitated labeling of recombinant CB_2_ with stable isotopes^4,8^. However, *E.* *coli* cells lack the machinery for co- and post-translational modifications such as glycosylation and palmitoylation that are known to play a role in maintaining structural stability, cellular trafficking, and functional activity of GPCR in cell membranes^9–13^. Here, we explore the feasibility of mammalian cell-based platforms for expression of functional CB_2_ receptor with native-like posttranslational modifications in large quantities. We further examine whether these modifications contribute to the stability of the purified receptor reconstituted in detergent micelles of defined composition. Last but not least, we demonstrate that our procedure allows for labeling of CB_2_ with ^13^C_5_-methionine to obtain samples for high resolution NMR spectrum analysis, which may be extrapolated to other GPCRs.

Several eukaryotic expression systems have been used for preparation of GPCR for structural studies including insect cells *Spodoptera* *frugiperda* *Sf9,* *Sf21* and *Trichoplusia* *ni* *(Tni*) High five^14–20^. The high-yield production of a modified sequence of CB_2_ in Sf9 cells was reported recently^21^. However, production of GPCR in insect cells, especially for subsequent NMR studies, has potential drawbacks: the growth medium is rather complex and contains undefined levels of amino acids and peptides. It is not suited for preparation of a medium depleted of specific amino acids, necessary for NMR experiments. An attempt to express CB_2_ in yeast cells was only partially successful since only a fraction of the expressed receptor was ligand-binding competent^22,23^.

In this work we report the expression of the wild type CB_2_ in mammalian cell cultures. An increasing number of successful examples of GPCR expression in mammalian cells have been published recently^14,24,25^. These include serotonin receptor 5HT3A expressed in tetracycline-induced HEK293S-TetR cells (about 1.7 mg/L of culture)^26^ and olfactory receptor 17-4 expressed in HEK293S-GNTI-cultivated in bioreactor (3 mg/L of culture)^27^. The highest reported production was that of rhodopsin in HEK293S-TetR cells in a bioreactor (9 mg/L)^28^. To our knowledge, there are currently no reports of high-level expression of recombinant cannabinoid receptors in mammalian cells.

Here, we describe the development of large-scale production of CB_2_ in Expi293F cells and its glycosylation-deficient derivative, Expi293F GNTI^−^ cell line. Expi293F cells are human cells derived from the HEK293F cell line, and are a core component of the Expi293F Expression System. They are maintained in suspension culture and will grow to high density in Expi293F Expression Medium. Expi293F cells are highly transfectable and generate superior protein yields compared to standard HEK293 cell lines in transient protein expression. Expi293F GNTI^−^ cells are derived from Expi293F and have been engineered to lack *N*-acetylglucosaminyl-transferase I (GnTI) enzyme activity leading to the production of glycoproteins with a uniform high mannose glycopattern.

Spectroscopic techniques such as nuclear magnetic resonance (NMR) require large quantities of purified and homogenous protein sample with sufficient stability over the duration of the experiment. Since GPCR are highly hydrophobic, they require solubilization in detergents or other solubilizing agents, at concentrations in the mid-to high-micromolar range for NMR analysis. The preferred small size of the protein-detergent particles imposes significant restrictions on detergent and lipid molecules comprising a micelle. So far, few recombinant GPCR and detergent systems satisfy these stringent requirements^29–31^. Current approaches to improve stability of the target receptors include thermostabilization of recombinant receptors by mutagenesis^32^, encapsulation of proteins by styrene maleic acid (SMA) co-polymer or similar polymers with retention of some annular lipids^33^, or reconstituting receptors in nanodiscs stabilized by scaffold proteins^29^.

Here, we sought to improve the stability of CB_2_ in detergents by taking advantage of the mammalian cell-expression system to produce recombinant GPCR with native-like co- and post-translational modifications (PTM), and combine it with the stabilization potential of Façade detergent^34^ to form small bicelle-like particles that encapsulate GPCR and lipids. We examine the contribution of both glycosylation and acylation of the recombinant CB_2_ receptor to its stability in detergents. We will further present evidence that untruncated CB_2_ stabilized via post-translational modifications and selectively labeled with ^13^C_5_-Met can be successfully analyzed by NMR. Our findings may provide a useful path for a robust preparation of milligram quantities of stable wild-typeGPCRs with native PTM for structural studies at conditions near physiological.

## Results

### Expression of CB_2_ in mammalian cell culture

CB_2_ receptor without post-translational modifications was expressed in *E.* *coli* as a N-terminal fusion with maltose-binding protein (MBP) followed by a TEV-protease recognition sequence and a twin-Streptag, and a His tag fused to the C terminus of CB_2_ (Fig. S1c) as described earlier^6^. Both the twin-Streptag and a histidine tag have been shown not to influence expression levels nor the activity of CB_2_ while the MBP fusion partner is essential for expression of a functional receptor in bacterial cells^3^. MBP was removed during purification of CB_2_, upon treatment with TEV protease.

For expression of CB_2_ with post-translational modifications, mammalian expression systems were used. The expression constructs are shown in Supplementary Fig. S1. A design of experiment (DOE) as described in Supplemental information was set up for optimization of expression conditions. Briefly, two cell lines were selected for expression trials: Expi293F and Expi293F GNTI^−^. The latter cell line was engineered to lack *N*-acetylglucosaminyltransferase I (GnTI) and therefore lacks complex *N*-glycans^28,35^. All expression experiments were performed in a 24 well deep well format following the protocols for transient transfection detailed in “Methods” section. Cell cultures were analyzed for CB_2_-GFP expression by FACS and viable cell density (VCD) measurements over a time span of 5 days. Since FACS analysis measures intracellular fluorescent signal on a per cell basis, we multiplied the geometric mean of fluorescent intensity (MFI) by the VCD as to estimate the total amount of harvestable CB_2_-GFP in a given volume of cell culture (Supplementary Figs. S2–S3). The initial DOE enabled us to narrow down conditions and test the addition of the stabilizing ligand CP-55,940 in the mammalian cell system since it was shown earlier to stabilize the receptor in bacterial cells^7^. We observed no toxicity of the ligand at 5 μM and CB_2_-GFP expression was more stable over time by addition of the ligand Supplementary Fig. S4a). According to VCD, the optimal harvest time did not change with supplementation (Supplementary Fig. S4b). Based on these observations the optimal conditions for CB_2_-GFP expression were determined to be: 100% Expi293 Enhancer 1, 100% Expi293 Enhancer 2, 1 μg/mL DNA per ml of culture to transfect. Optimal harvest time was 48 h post transfection for both systems.

To verify the validity of our CB_2_-GFP fusion model, we employed a FACS based assay to measure CB_2_ and CB_2_-GFP at the plasma membrane using a monoclonal anti-CB_2_ antibody raised to the extracellular N terminus of CB_2_. We observed that expression of either CB_2_-GFP or the native CB_2_ receptor when measured at the plasma membrane peaked at 3 days post transfection in all cell systems (Supplementary Fig. S5). There was no significant difference between the accumulation of CB_2_ or the CB_2_-GFP fusion protein in either of the Expi293F derived cell systems, although Expi293F GNTI^−^ cells appeared to have a higher amount of either CB_2_ construct at the plasma membrane than the maternal Expi293F cells. These results validated the use of a GFP-tagged CB_2_ receptor as an initial model for expression optimization in our Expi293F based systems.

**Figure 1:**
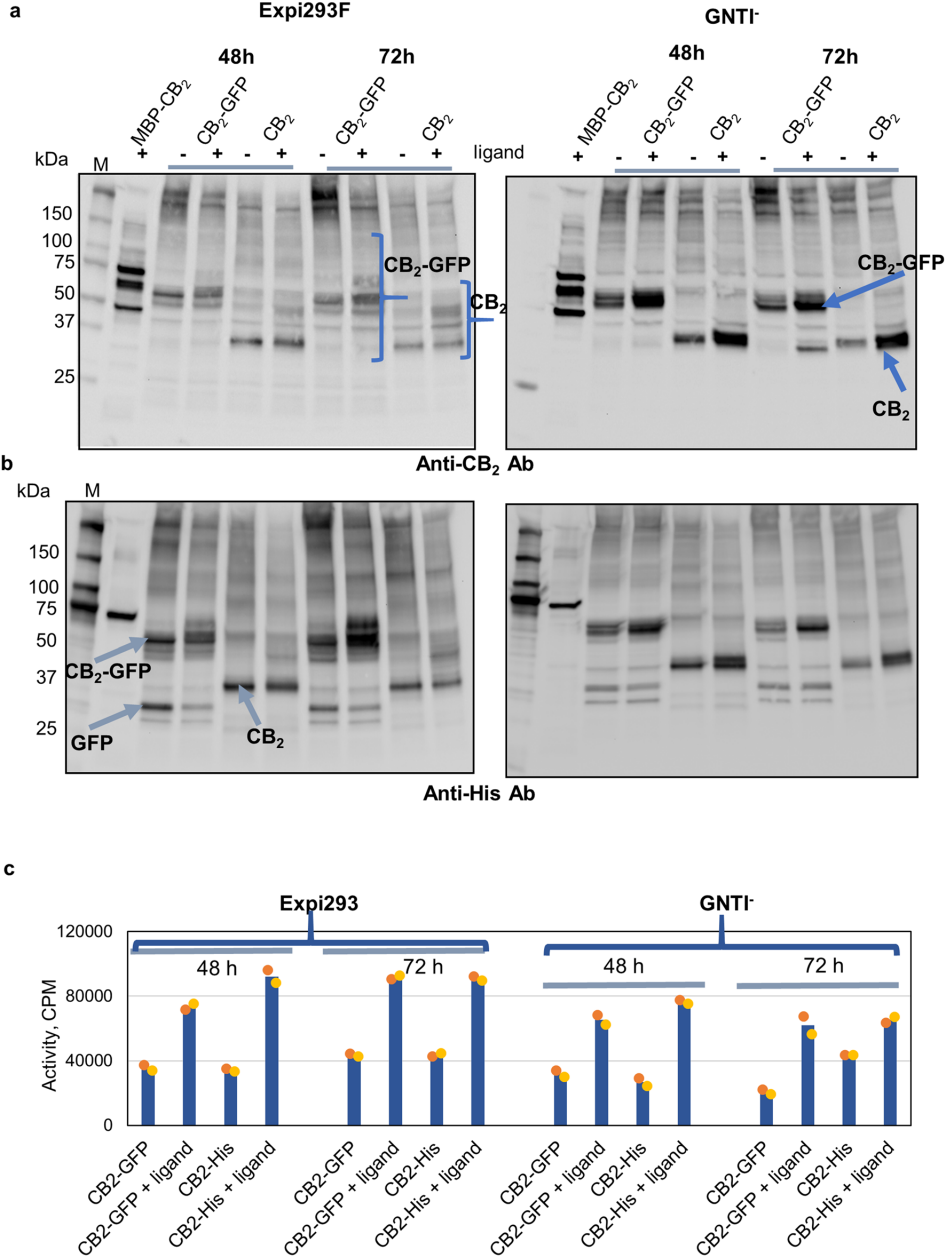
Optimization of expression of CB_2_ in Expi293F and Expi293F GNTI^−^ cells. Cells were collected at 48- and 72-h post-transfection, and membrane preparations obtained as described in “Methods”. (**a**) Western blot detection with anti-CB_2_ antibody and (**b**) with anti-His-tag antibody. 20 μg of membrane protein per lane. (**c**) activity of CB_2_ in membranes measured by G protein activation assay, 2 μg protein per reaction. Bars represent an average of two independent measurements with individual data points shown by dots.

To validate FACS data, cell membrane fractions were analyzed for expression of CB_2_-GFP and CB_2_ constructs by Western blot (Fig. 1a,b) followed by the activity tests on CB_2_ in membrane preparations (Fig. 1c). Since this analysis needs more biomass, cells were transfected in a 30 mL shake flask format. As there was some uncertainty about the optimal time of harvest arising from the data described above, biomass was harvested at 48 and 72 h post transfection. We observed a good correlation between the expression of the tagged CB_2_-GFP and un-tagged CB_2_ in Expi293F and Expi293F GNTI^−^ cells. The accumulation of the recombinant protein in whole cell membrane preparations was the highest at 48 h post-transfection.

Addition of the high affinity agonist CP-55,940 increases levels of expression of recombinant CB_2_ in *E.* *coli* cells^36^. We therefore tested if a similar effect could be observed in mammalian systems. Indeed, in both Expi293F and Expi293F GNTI^−^ cell lines, the addition of CP-55,940 increased the levels of CB_2_ in membrane preparations (Fig. 1a,b). The expression in Expi293F GNTI^−^ cells produces homogenous preparations of CB_2_ protein as opposed to Expi293F cells that express several glycosylated species of CB_2_. At the same time, the Expi293F cells seem to produce slightly higher levels of active receptor. In order to obtain high quality, homogenous protein preparations for subsequent biochemical and biophysical studies, we proceeded with large-scale CB_2_ expression in the Expi293F GNTI^−^ cell line.

### Assessment of CB_2_ thermostability in membrane preparations

**Figure 2:**
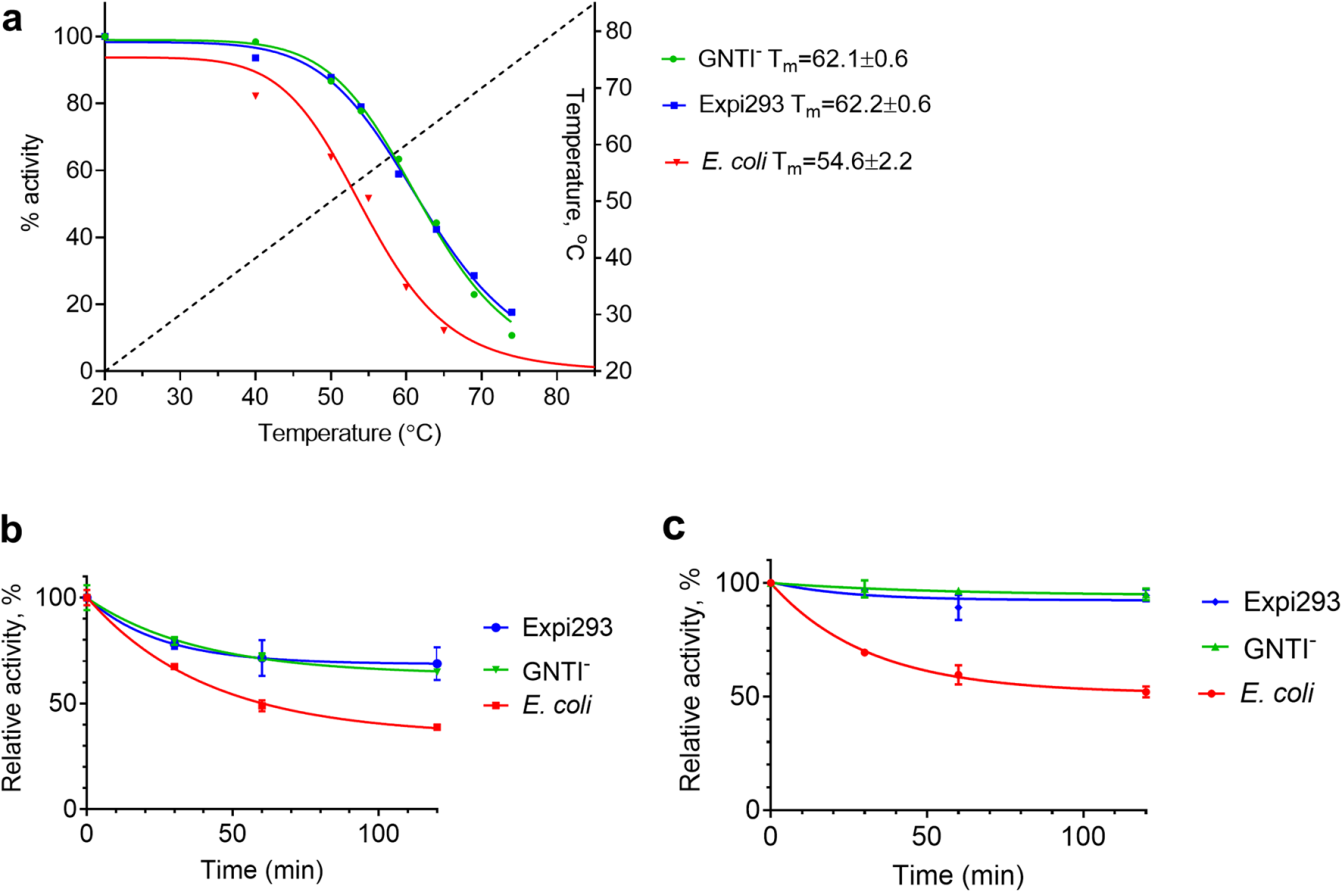
Thermostability of CB_2_ in membranes from different cell lines. (**a**) Temperature ramp 1 °C/min, temperature gradient shown by dotted line. 2 μg membrane protein per sample (**b**), membranes were incubated at 42 °C without ligand; (**c**) membranes were pre-treated with 5 μM CP-55,940, then incubated at 42 °C and aliquots withdrawn at time intervals indicated. Results of duplicate measurements determined by G protein activation test are presented.

Expression in mammalian cell lines produces recombinant receptor with such co- and post-translational modifications (PTMs) that cannot be attained in an *E.* *coli* expression system. Here, we explored whether these PTMs may contribute to improved stability of the receptor. Thermostability of receptor in membrane preparations was assessed by analyzing its functional activity upon exposure to elevated temperatures. Functional activity of receptor was measured by quantifying the rates of activation of cognate G protein in an in vitro assay, as detailed in “Methods”. Isothermal stability of CB_2_ was measured by incubating membrane preparations at 42 °C. Temperature of unfolding (or apparent “melting” temperature) was measured upon subjecting membrane preparations to a gradient of temperature. Aliquots were withdrawn at indicated time intervals, and the residual activity of the receptor analyzed by quantifying the rates of G protein activation (Fig. 2a).

CB_2_ in Expi293F cell membranes was more stable (T_m_ = 62 °C) than its counterpart in *E.* *coli* BL21 (DE3) membranes (T_m_ = 54 °C). The difference in glycosylation patterns of recombinant receptors expressed in Expi293F and Expi293F GNTI^−^ cells (Fig. 1a) does not seem to affect their stability. The isothermal stability of CB_2_ in Expi293F membranes was much higher than in *E.* *coli* cell membranes at 42 °C (Fig. 2b). Addition of high affinity ligand to membranes greatly improved the stability of CB_2_ receptor: less than 5% of initial activity was lost after a 2-h incubation at 42 °C. The receptor in *E.* *coli* membranes was much less stable at these conditions, losing about 50% of its initial activity (Fig. 2c).

### CB_2_ purification

Besides PTMs, there are multiple factors influencing stability of GPCR in membranes, including lipids and other membrane proteins. Solubilization in detergents and chromatographic purification removes other proteins, as well as the bulk of membrane lipids. However, covalent PTM of CB_2_ are expected to be preserved. We hypothesized that these modifications may improve the stability of CB_2_ not only in membranes but also in detergent micelles. Therefore, the stability of recombinant receptor, purified from Expi293F and Expi293F GNTI^−^ was compared to CB_2_ from *E.* *coli* cells.

**Figure 3:**
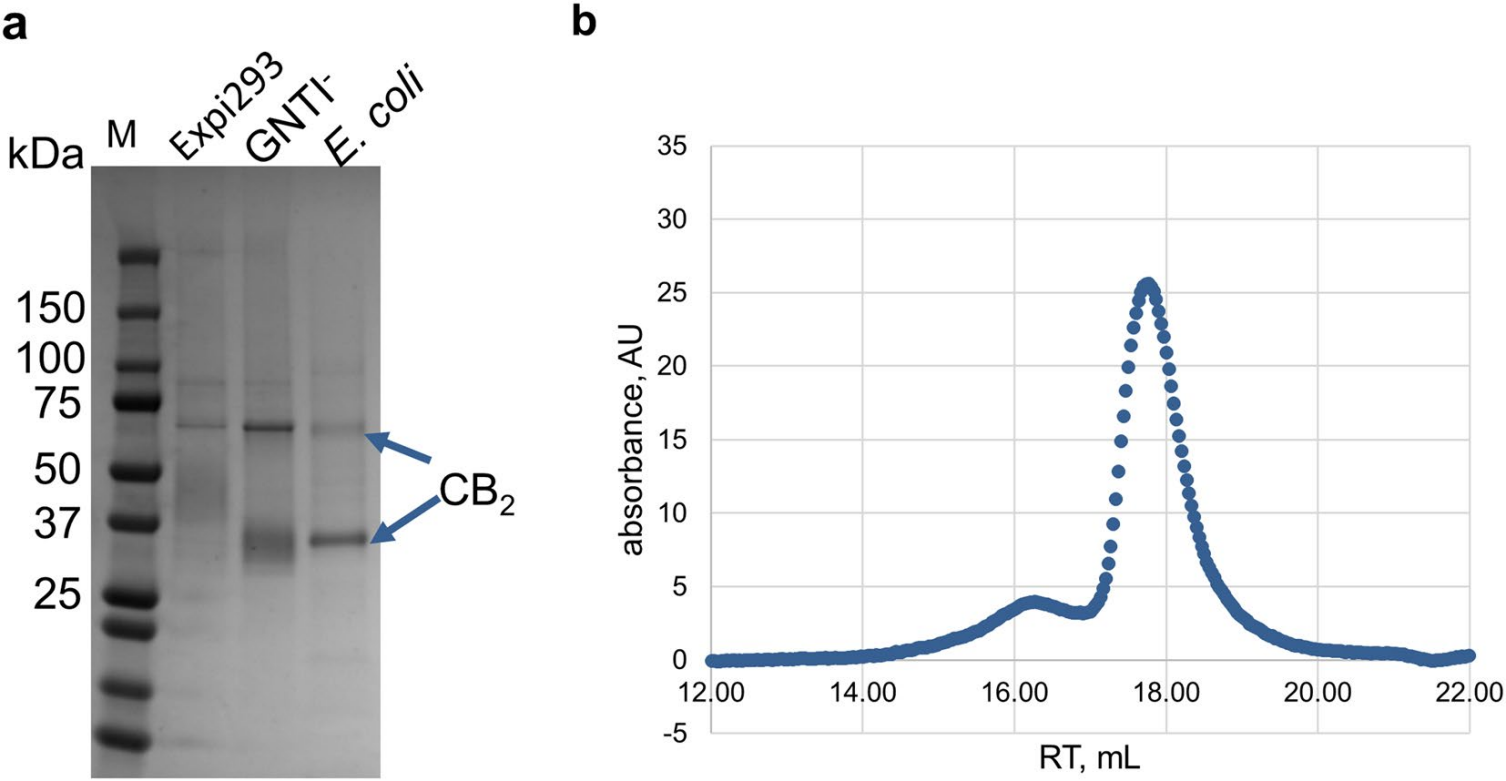
Purified CB_2_ from Expi293F, Expi293F GNTI^−^ and *E.* *coli* BL21(DE3) cells. (**a**) SDS-PAGE (4–20%) stained with Instant Blue. 1.5 mg of purified protein per lane. Monomer and dimer forms of CB_2_ are indicated by arrows. Dimers and higher oligomers of CB_2_ are typically formed at conditions of SDS-PAGE^5,37^. (**b**) Size-exclusion chromatography of CB_2_ purified from Expi293F GNTI^−^ cells. Superose Increase 6 (10/300) column; 50 mM Tris–HCl pH 7.5, 100 mM NaCl, 0.25 mM Façade-TEG/0.025 mM CHS.

CB_2_ receptor was purified from Expi293F and *E.* *coli* expression cell lines by two successive rounds of affinity chromatography as described in “Methods”. A typical purification from Expi293F GNTI^−^ cells is illustrated in Supplementary Fig. S6. The protein was purified in the presence of CP-55,940 in mixed Façade-TEG micelles with addition of a derivative of cholesterol, cholesteryl hemisuccinate (CHS). Both ligand and CHS are known to greatly improve the stability of the receptor^34^. Similar procedures were employed for purification of CB_2_ from Expi293F cell and *E.* *coli* BL21(DE3) cells (not shown). Purified receptor, solubilized in Façade-TEG/CHS micelles (Fig. 3a), was predominantly monomeric as demonstrated by size-exclusion chromatography (Fig. 3b). NMR analysis of protein-harboring micelles revealed that in addition to Façade-TEG and CHS, they also contain some residual phospholipid extracted from cell membranes and carried over during chromatographic purification (Supplementary Fig. S7).

About 2 mg of purified CB_2_ was recovered from 1L of Expi293F GNTI^−^ cell culture as well as from Expi293F cells (not shown). Such yield, per unit of cell culture volume, is higher than what was reported previously for expression of functional CB_2_ in shake flasks of *E.* *coli* BL21(DE3) cells^5,6^ (0.5–1 mg/L), and is comparable to the yield of CB_2_ protein obtained by high-density fermentation of the *E.* *coli* in a fermenter under controlled condition including pH, temperature and oxygenation (2 mg/L)^8,37^. These results indicate feasibility of using a mammalian cell expression system for large-scale preparation of CB_2_.

### Thermostability assessment

Thermostability of purified CB_2_ protein was assessed in Façade-TEG/CHS detergent micelles as described in “Methods”. The choice of detergent system was based on our recent findings that Façade-TEG is equal to or even superior to other commonly used non-ionic detergents such as dodecylmaltoside in its ability to stabilize the GPCR in a soluble, monomeric form^34^. It has been proposed that Façade-TEG detergent forms a small bicelle-like structures that accommodate CHS and lipids that co-purify with the receptor^8,38^. Here, we measured the micelle size in diffusion experiments conducted in D_2_O (Supplementary Figs. S8, S9) and Supplementary Table S1. The hydrodynamic radius of the empty Façade-TEG micelle at 1 mM concentration was 1.45 ± 0.02 nm and for CB_2_-conatining micelle—4.90 ± 0.06 nm. The CB_2_ preparation contained a fraction of particles of large size.

**Figure 4:**
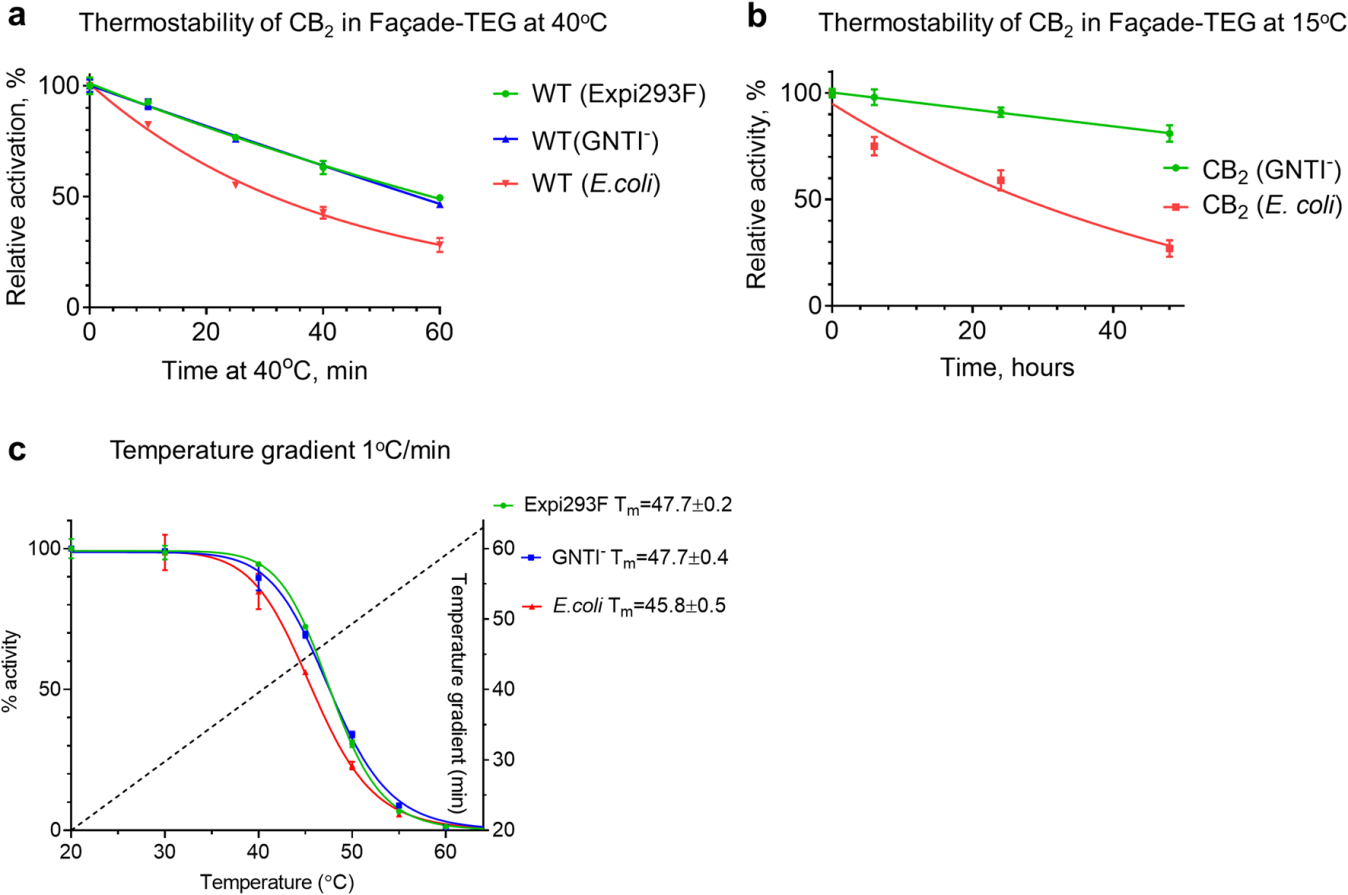
Thermostability of purified CB_2_ proteins in 0.25 mM Façade/0.025 mM CHS/10 μM CP-55,940. Equivalent amounts of protein (20 ng per reaction) were taken. Each point represents an average of duplicate samples with errors indicated. (**a**) Incubation at 40 °C; (**b**) incubation at 15 °C; (**c**) temperature ramp of 1 °C/min. Temperature gradient shown by dotted line.

The isothermal stability of CB_2_, isolated from Expi293F, Expi293F GNTI^−^ and *E.* *coli* BL21 (DE3) cells was assessed on protein-containing micelles at either 40 °C (Fig. 4a) or 15 °C (Fig. 4b). The apparent melting temperature of the protein was measured at a temperature ramp of 1 °C/min (Fig. 4c).

CB_2_ isolated from Expi293F and Expi293F GNTI^−^ cells exhibited higher isothermal stability at 40 °C compared to the bacterially expressed receptor (Fig. 4a). Likewise, the T_m_ of HEK cell-produced protein in micelles was reproducibly higher than that of *E.* *coli*-expressed protein. There was no substantial difference between the stability of proteins isolated from Expi293F and Expi293F GNTI^−^ cells despite differences in glycosylation patterns of CB_2_. These results suggest that the modifications attained by expression in mammalian cell lines may contribute to better stability of CB_2_ in detergents.

Importantly, the activity of receptor isolated from Expi293F GNTI^−^ cells declined only slightly (18%) after two-day incubation at 15 °C while the protein from *E.* *coli* cells lost almost 80% of its activity. The higher stability of the mammalian cell-expressed receptor in Façade detergent enables such applications as solution-state NMR that require longer stability of protein samples.

### Post-translational modifications and stability of CB_2_

Understanding the reasons for higher thermostability of CB_2_ isolated from mammalian cells may be particularly important for efficient expression strategies for preparation of stable, functional GPCR for all types of structural studies. We focused on glycosylation and palmitoylation of CB_2_ as the two likely contributing factors.

#### Glycosylation

Putative glycosylation sites on CB_2_ were analyzed by LC/MS/MS after trypsin digestion of the purified protein sample with or without treatment by PNGase F, an amidase that cleaves between the innermost GlcNAc and asparagine residues of the protein as described in “Methods” (Supplementary Fig. S10). CB_2_ contains only one conserved N-linked glycosylation motif (NXT/S, N_11_GS). The de-amidation of Asn11 (Supplementary Fig. S11) suggests that it represents a glycosylation site, consistent with expected removal of a glycan by PNGase F. The peptide containing Asn11 was not detected in CB_2_ preparation without deglycosylation treatment. The glycan form on Asn11was not determined in this study although the available evidence for expression of other recombinant proteins in GnTI-deficient cell lines suggests the predominant formation of Man_5_GIcNAc_2_*N*-glycans^39,40^.

**Figure 5:**
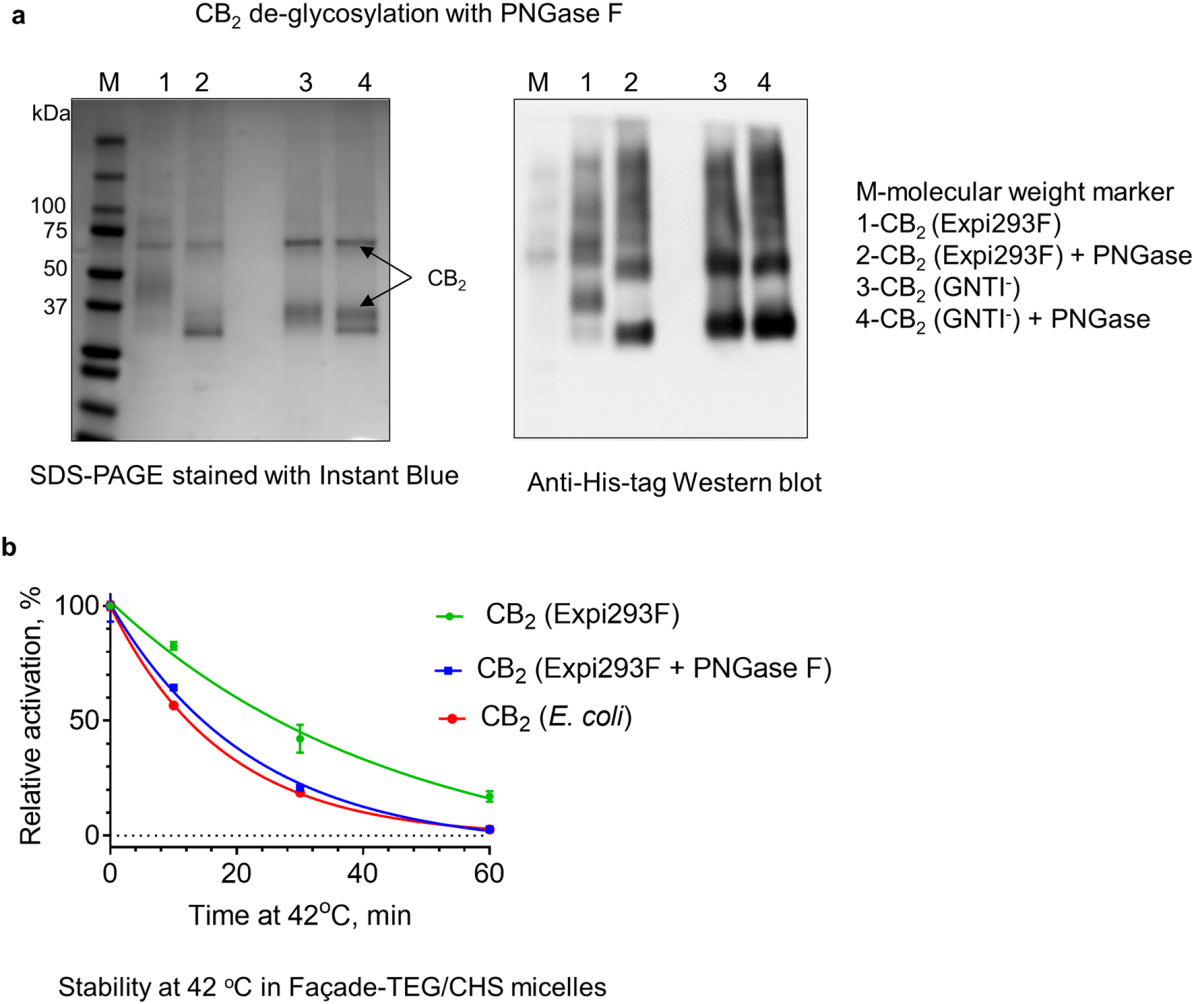
Effect of PNGase F treatment on stability of purified CB_2_ protein. (**a**) SDS-PAGE and Western blot of untreated and treated proteins purified from Expi293F and Expi293F GNTI^−^ cells. (**b**) Thermostability of untreated and treated protein samples at 42 °C measured by G protein activation. CB_2_ purified from *E.* *coli* BL21(DE3) serves as a control.

To obtain further insight into the effect of glycosylation on stability of CB_2_ protein in detergents, we enzymatically removed the N-linked oligosaccharides by PNGase F cleavage (Fig. 5a). As expected, such treatment results in a decrease of the apparent molecular weight of CB_2_ isolated from both Expi293F and Expi293F GNTI^−^ cells.

The PNGase F-treated protein from Expi293F™ was less thermostable than its untreated variant (Fig. 5b). Similar results were obtained for CB_2_ purified from Expi293F GNTI^−^ cells (not shown). For comparison, the temperature inactivation curve of the un-glycosylated protein from *E.* *coli* is shown. Therefore, the glycosylation of CB_2_ is associated with increased thermostability.

### Acylation and stability

S-linked palmitoylation as well as other types of acylation have been implicated in targeting GPCR to specific cellular compartments, stabilization in membranes, and modulating functional activity^10,11,41,42^. Acylation typically occurs at the C terminus of the receptor although modifications of intracellular loops linking transmembrane domains of the receptor have also been reported^42^. The C terminus of CB_2_ contains three cysteine residues, in positions 313, 320, and 360 (Supplementary Fig. S11). To examine whether any of these residues is targeted by acylation we expressed cysteine-deficient variants of the receptor in Expi293F and Expi293F GNTI^−^ cells replacing cysteines 313, 320 and 360 with a serine residue, one at a time. In a construct termed 3-MUT, all three residues were replaced simultaneously. The constructs C313S, C360S and 3-MUT were expressed in Expi293F GNTI^−^ cells at about the same level as the wild type (WT) receptor while C320S was expressed at lower levels (Supplementary Fig. S12a). All constructs showed functional activity as determined by activation of G protein (Supplementary Fig. S12b). The replacement of Cys residue in position 320 appears to have the greatest effect on stability of receptor, shifting the apparent T_m_ value from 72.9 min (WT) to 64.5 min while mutations in positions C313 and C360 did not have any significant effect (Supplementary Fig. S13a). The construct harboring all three mutations (3-MUT) exhibited a T_m_ value similar to that of the C320S. Similar results were obtained for membrane samples from Expi293F cells (not shown). Modifications at C320 may contribute to CB_2_ stability in membranes. Alternatively, the replacement of cysteine in this position may disrupt the formation of homodimeric CB_2_ which could have higher stability. However, no evidence for homodimers of CB_2_ in cell membranes has been reported so far. Also, preliminary results from NMR experiments on micelles suggest that the majority of HEK cell-expressed CB_2_ is monomeric.

**Figure 6:**
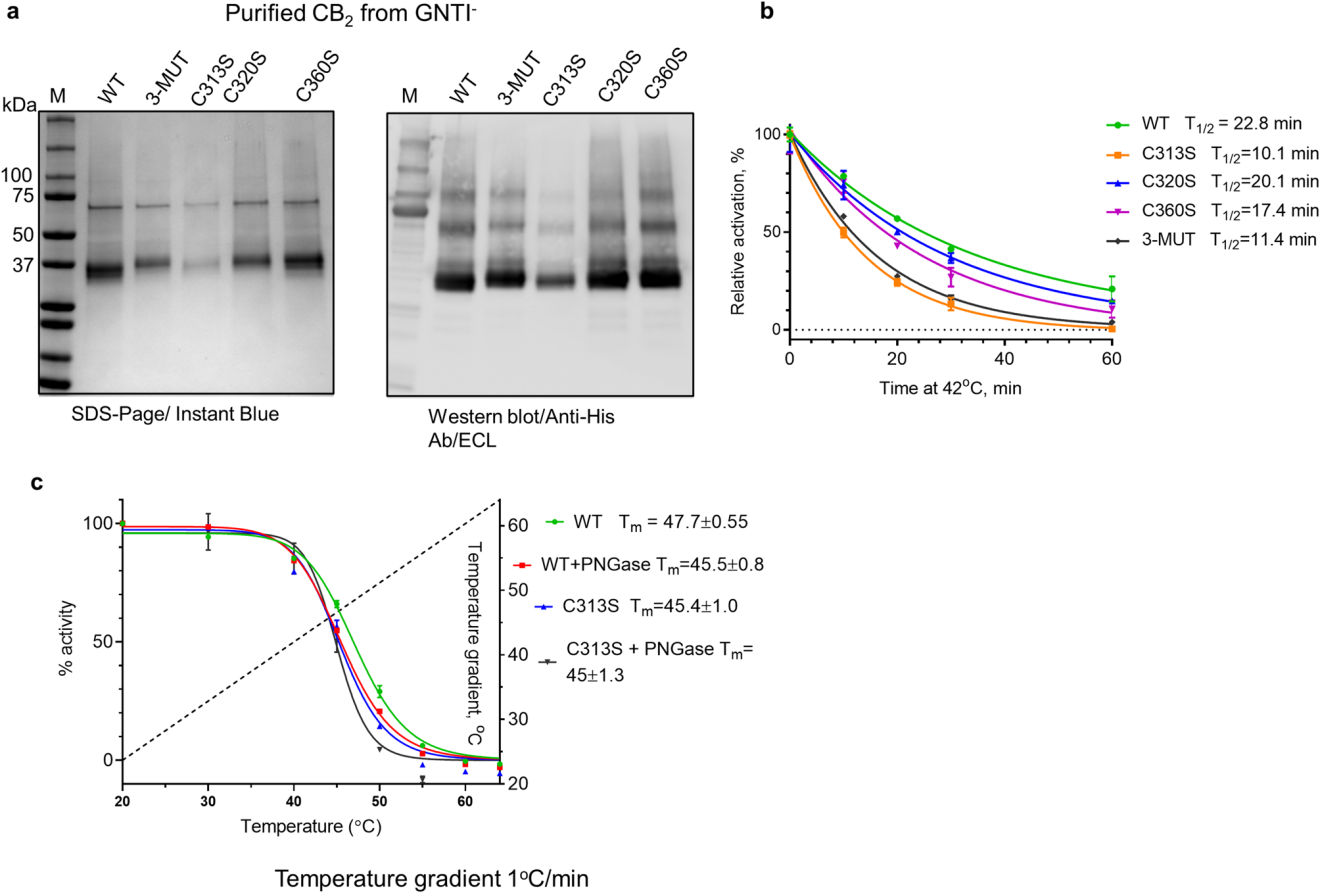
Purification and thermostability of CB_2_ WT and cysteine-replacement variants. (**a**) SDS-PAGE and Western blot of purified CB_2_ variants from Expi293F GNTI^−^ cells; (**b**) thermostability at 42 °C of CB_2_ WT and cysteine replacement variants in Façade-TEG/CHS micelles; (**c**) contribution of glycosylation and palmitoylation into stability of CB_2_. Treatment with PNGase F removes N-linked glycosylation from the receptor while replacement of cys in position 313 removes site for palmitoylation. Results represent an average of two independent measurements with standard deviations indicated.

The cysteine replacement constructs were purified from Expi293F GNTI^−^ cells (Fig. 6a) and their thermostability compared to that of the wild type. The stability of C320S and C360S was only slightly lower than that of the WT while the stability of C313S and 3-MUT decreased significantly (Fig. 6b). In fact, the stability of C313S was as poor as that of the WT CB_2_ obtained from *E.* *coli* cells (Supplementary Fig. S13b). Therefore, our data suggest that C313 is a site of a post-translational modification that may contribute to the stability of CB_2_.

We studied the PTM at C terminus of CB_2_ by subjecting the purified WT and three cysteine replacement constructs to tryptic digest followed by LC/MS/MS analysis (Supplementary Fig. S15). The WT CB_2_ and the construct C360S were found to be partially palmitoylated at position C313S (Supplementary Figs. S15, S16) while the C313S construct was not. No palmitoyl residue was observed at positions C320 and C360 on any of the constructs analyzed (Supplementary Figs. S17–S20). S-linked palmitoylation is known to be a reversible process, and the palmitoyl moiety can be partially lost depending on the treatment of the protein^43,44^. Our results suggest that C313 is targeted by acylation and the absence of this PTM correlates with lower stability of the purified protein in detergent micelles.

Furthermore, our results suggest that both N-terminal and C-terminal modifications of CB_2_ may contribute to stability of this receptor. To confirm this, we subjected both the WT and C313S variant of CB_2_, isolated from GNTI^−^ cells, to treatment with PNGase F and studied the thermostability of these proteins by applying a temperature gradient (Fig. 6c). The untreated WT exhibited the highest stability, C313S treated with PNGase had the lowest stability, while the WT treated with PNGase F, and the untreated C313S mutant exhibited intermediate stability. Thus, both the glycosylation of the N-terminal part of CB_2_ and palmitoylation of its C-terminal tail stabilize the mammalian cell-expressed receptor in detergents.

**Figure 7:**
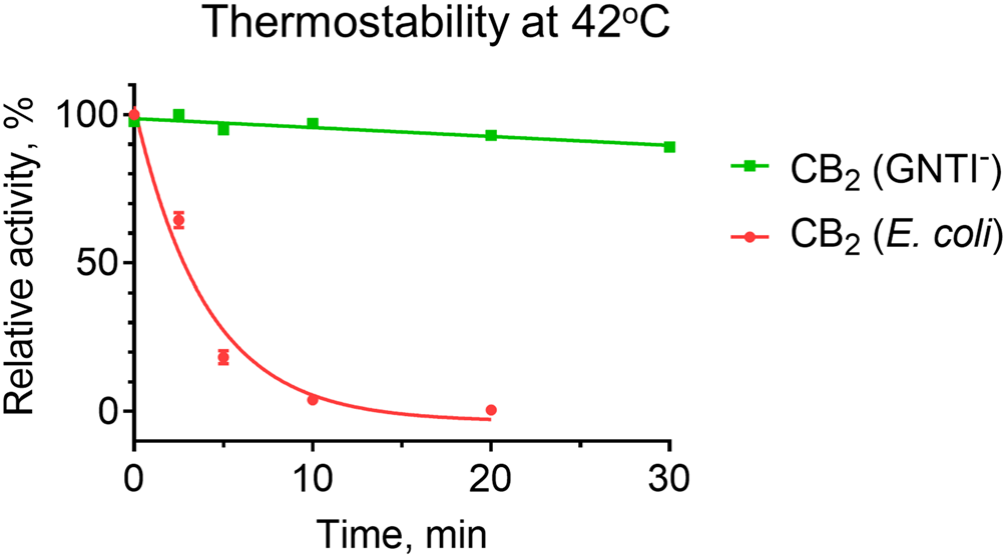
Stability of CB_2_ in mixed Façade-TEG/CHS/CHAPS micelles. Stability of purified proteins was measured by G protein activation as described in “Methods”. Results of duplicate measurements are presented.

It is well documented that the composition of detergent micelles affects the stability of solubilized GPCR^36,38,45^, and CHS is among the strongest stabilizing components in micelles^36^. Since the amount of CHS that can be dissolved in Façade detergent is limited (unpublished observations), we explored ways to increase the relative content of CHS in micelles hoping to achieve even greater stability of CB_2_. The purified CB_2_ in Façade-TEG/CHS (10:1, mol/mol) was supplemented with a mixture of CHAPS and CHS to the following concentrations of components: Façade-TEG-0.25 mM; CHAPS-0.5 mM; CHS-0.16 mM. Because of the relatively high critical micelle concentration of CHAPS (8 mM), this detergent is not expected to form micelles on its own. The stability of CB_2_ purified from Expi293F GNTI^−^ was measured in this modified detergent buffer (Fig. 7). Indeed, almost 100% of activity of CB_2_ purified from GNTI^−^ cells, was preserved upon 30 min incubation at 42 °C.This suggests even greater potential to improve the stability of the recombinant GPCR by combining PTM introduced by the expression host with judicious design of detergent-lipid composition of solubilizing micelles (compare with Fig. 4a).

**Figure 8:**
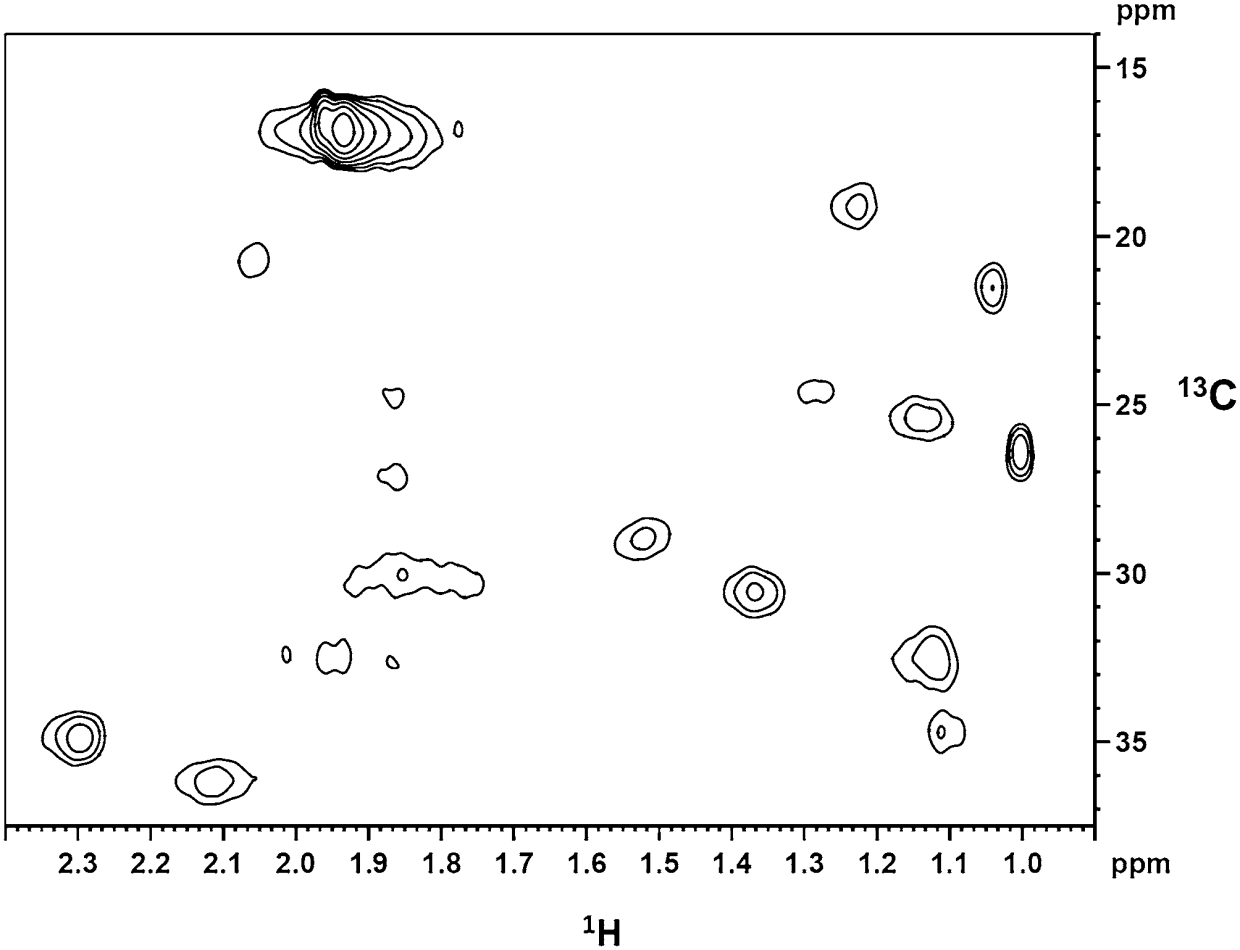
^1^H-^13^C HSQC spectrum of methionine-^13^C_5_-labeled CB_2_ in Facade-TEG/phospholipid/CHS micelles recorded at 15 °C. The selected spectral region shows the sidechain resonances of ^13^C-labeled methionine. Assignment was by comparison with a spectrum of unlabeled CB_2_. C_β_:1H (2.30 ppm, 2.12 ppm; ^13^C:35.0 ppm, 36.5 ppm), C_ε_: 1H: 1.9 ppm; ^13^C: 17.0 ppm).

We further analyzed CB_2_ protein metabolically labeled in Expi293F GNTI^−^ cells with ^13^C_5_-methionine by solution state NMR. The ^1^H/^13^C resonances originating from the side chains of the ten labeled methionine residues in CB_2_ sequence (Supplementary Fig. S21) were detected at decent resolution (Fig. 8). It confirms that the protein is solubilized in small micelles that are stable during the acquisition time of 20 h at 15 °C.

## Discussion

Here, we describe a robust methodology for high-level expression and purification of the human cannabinoid receptor CB_2_ derived from a mammalian cell culture. Highly functional CB_2_ receptor was produced by the Expi293F system. A more homogenous protein preparation, likely due to a more uniform glycosylation pattern was obtained by expression in the Expi293F GNTI^−^ system. Purified protein yields from both cell systems are about 2 mg/L of culture. This is among the highest expression rates reported for recombinant, full-length GPCR, other than rhodopsin, expressed in mammalian cells^14,25^. We further demonstrate that the protein can be metabolically labelled with ^13^C_5_-methionine at high efficiency, with the same yield as the unlabeled receptor. The CB_2_ receptor prepared by expression in Expi293F GNTI^−^ cells exhibited significantly higher stability in Façade detergent than its bacterially expressed counterpart. This enables characterization of functional cannabinoid receptor by high-resolution NMR.

Initial optimization of CB_2_ expression conditions relied on expression of CB_2_-GFP fusion protein quantified by FACS. Our results suggest that for CB_2_, GPCR-GFP fusion proteins can be a useful approach for optimization of initial expression conditions. The validity of this approach appears to depend on the type of the cellular expression system and may further vary when assessing optimal expression of other GPCRs. It is not known yet if experience is transferable to expression of other GPCR. Our data demonstrate that it is advisable to complement internal cell fluorescence analysis by measuring the relative level of receptor expression in the membrane fraction of interest. For CB_2_, expression in a whole cell prep peaked at 48 h post transfection, whereas the CB_2_ accumulation in the plasma membrane peaked 24 h later. Furthermore, functional activity needs to be confirmed to ensure the quality of the GPCR of interest. Taking all of the above into account, small scale expression of CB_2_-GFP in Expi293F expression systems provided relevant guidelines for larger scale high yield expression runs needed to obtain the necessary amount of purified CB_2_ receptor to perform NMR studies. We believe that our study may provide a useful guidance for optimization of high-level expression of GPCR in mammalian suspension cell culture.

Expression of GPCR in mammalian cell lines results in a native-like pattern of co- and post-translational modifications. In the case of CB_2_, we demonstrate that the asparagine residue in position 11 of its N terminus is targeted for N-linked glycosylation. The C-terminal part of the receptor undergoes *S*-acylation in position Cys313. Combined with reconstitution in Façade-TEG detergent, these modifications greatly improve the thermostability of CB_2_. Both Expi293F and its glycosylation-restricted variant Expi293F GNTI^−^ result in higher stability of the expressed protein in detergent micelles. To our knowledge, effects of glycosylation on stability of GPCR in detergent micelles have not been previously reported. It may be of interest to point out that the flexible N-terminal domain of GPCR is often truncated to improve formation of homogenous protein crystals for structural studies. However, this may impact structural properties of the protein. Therefore, for spectroscopic studies of GPCR, it would be desirable to preserve the N terminus of the receptor.

Here we demonstrated that glycosylation of the N-terminal part of CB_2_ contributes to higher stability of the protein in detergents. It would be of interest to investigate whether glycosylation contributes to stability of other recombinant GPCR as well.

The C-terminal part of CB_2_ plays an important role in its stability. We demonstrated that Cys313 is at least partially palmitoylated. The Cys313 residue was proposed to participate in formation of helix 8, proximal to TM and part of C terminus of CB_2_^46^. The palmitoyl residue at C313 may interact with the hydrophobic fraction of the bicelle formed by lipids, CHS and Façade detergent, thereby reducing conformational flexibility of the receptor.

Our results show that position C320 in the purified CB_2_ is unlikely to be palmitoylated, and the stability of C320S is the same as for WT CB_2_ in Façade detergent. On the other hand, C320S as well as the triple mutant 3-MUT exhibited noticeably lower stability in Expi293F membranes. While the mechanism of this effect is unclear, it is possible that this cysteine residue may engage in a formation of a disulfide bridge with the corresponding residue of an adjacent molecule of CB_2_. If such interaction indeed takes place, it may result in a higher stability of the resulting dimer. However, we cannot exclude the possibility that C320 is palmitoylated in cells as well. Since acylation of GPCR in cells is a highly dynamic process, such modifications may not be easily detectable by mass-spectrometry methods.

We reported a successful expression of CB_2_ as a fusion with the maltose binding protein in *E.* *coli* previously^7,37^. The Façade detergent forms bicelle-like particles that incorporates the stabilizing CHS and lipids that co-purify with the recombinant protein^38^. Such protein/detergent/lipid particles are suitable to study the functional properties of CB_2_ as well as its structural characteristics^5,34,37,47^.

Finally, we demonstrated that supplementation of Façade-lipid-protein bicelles with addition of cholesterol derivative CHS improves thermal stability of mammalian-cell expressed CB_2_ on top of all other measures. Higher stability is of practical importance for all structural studies of GPCR.

## Methods

### Chemicals and reagents

Oligonucleotides were purchased from Operon Biosciences. Restriction enzymes and DNA-modifying enzymes were obtained from New England Biolabs. The Ni-NTA resin was from Qiagen. The StrepTactin XT Superflow was IBA GmbH. Mouse monoclonal antibodies against 6x-His tag were from ThermoFisher Scientific (Cat No MA1-21315). Mouse monoclonal antibody against GFP were from Invitrogen (Cat No GF28R, MA5-15256). Monoclonal antibody against human cannabinoid receptor CB_2_ was from R&D Systems (Cat No MAB36551-10 or FAB36551R). Mouse monoclonal antibody against Streptag were from IBA Life Sciences (Cat No 2-1507-001). Secondary ECL anti-mouse IgG from sheep conjugated with horseradish peroxidase were from GE Healthcare (Cat No NA931). Cholesteryl hemisuccinate Tris salt (CHS) and detergents 3 [(cholamidopropyl) dimethylammonio]-1-propanesulfonate (CHAPS) and n-dodecyl-β-d-maltoside (DDM) were obtained from Anatrace. Façade-TEG detergent was purchased from Avanti Polar Lipids Inc. Synthetic cannabinoid ligand CP-55,940 was from Cayman (Cat No 90084). ^13^C_5_-methionine was from Cambridge Isotopes (Cat No CLM-893-H-MPT-PK).

Expi293F cells (Cat No A14635) and Expi293F GNTI^−^ cells (A39240), expression media and transfection kits (Cat No A14635), and the Methionine labeling kit (Cat No A41249) were from ThermoFisher Scientific.

All other chemicals of reagent grade were purchased from Sigma.

### Expression constructs

Constructs for expression of CB_2_ in mammalian cells were based on accession number ENSG00000188822. Non optimized gene blocks were synthesized and cloned into pCDNA3.4 expression vector by GeneART (ThermoFischer Scientific). Cysteine-replacement variants of CB_2_ were synthesized by GenScript. Purified DNA plasmid for transfection was obtained from GeneArt (Supplementary Fig. S1a,b). The construct CB_2_-130 (Supplementary Fig S1c) was previously described^3^.

### Expression of CB_2_ in *E.coli*

CB_2_ was expressed as a fusion with the N-terminal MBP and affinity tags in *E.* *coli* BL21 (DE3) cells cultivated in 2xYT medium supplemented with glucose and ampicillin as described previously^5^. Cells were collected by centrifugation, washed with phosphate-buffered saline, and stored at − 80 °C. Membranes containing CB_2_ protein were prepared as described previously^3,48^, the expression levels of the protein determined by semi-quantitative Western blot, and the functional activity of CB_2_ measured by a G protein activation assay as described previously^49^.

### Expression of CB_2_ in mammalian cells

For expression of CB_2_ in mammalian cells, the native sequence of human CB_2_ gene was placed in the pCDNA3.4 vector (Supplementary Fig. S1a). To facilitate the early-stage optimization of expression methods, we used a C-terminal GFP-His tagged CB_2_ construct that could be easily detected by measuring whole cell fluorescence (Supplementary Fig. S1b). For purification and Western-blot detection and purification of the protein, a twin-Strep-tag was inserted at the N terminus, and a 6-His-tag was inserted at the C terminus of the construct. Expi293Fand Expi293F GNTI^−^ cells were cultured and transfected as per manufacturers protocol (MAN0007814). Cell viability, density and diameter were routinely determined on a Vi-cell XR cell counter (Beckman Coulter). To assure accuracy, samples were diluted 1:5 in growth medium before counting.

To determine the optimal expression levels of CB_2_, design of experiment (DOE) techniques were applied using various combinations of DNA, Enhancers and Feed as described. The DOE was performed in Axygen 24-well Clear V-Bottom deep well plates (P-DW-10ML-24-C-S). Briefly, on the day of transfection Expi293F cell cultures were diluted to 3 × 10^6^ cells/mL and 2.5 mL suspension was added per well. 8 μL ExpiFectamine 293 reagent was added to 140 μL Optiplex and incubated for 5 min before addition to 150 μL Optiplex containing 2.5 μg plasmid DNA. The complexation reaction mixture was further incubated for 10 min before addition to the cells. When used, stabilizing ligand was added at 5 μM directly after transfection. Cells were incubated for 18 h at 37 °C, a relative humidity ≥ 80% and a CO_2_ concentration of 8%, while shaking at 225 rpm on a 19 mm orbital shaker. Then, transfection enhancer 1 and 2 were added to the culture at the fractions described; 100% are equivalent to 15 μL and 150 μL for enhancer 1 and 2, respectively.

Cell cultures were further incubated at 37 °C, ≥ 80% relative humidity and 8% CO_2_ on the 19 mm orbital shaker until an aliquot was taken for analysis.

Cell cultures intended for preparation of membrane fractions were grown in a 125 mL flask at a final volume of 30 mL and harvested as described. For production on a larger scale, cells were transfected in a 3L corning flask and harvested as described. Methyl-^13^C-methionine labeling of CB_2_ was performed as described in the manual. The Gibco protein expression Calculator^50^ was used to calculate requirements for scaled-up production of protein. Additional information for the Expi293F system can be found online^51^.

### Detection of CB_2_ in cell cultures

Intracellular expression of CB_2_-GFP was assessed with an Attune NxT Flow Cytometer (ThermoFisher) equipped with an autosampler to collect samples at indicated time points. Signals from cellular debris were gated out by FSC/SSC gating and the geometric mean of the remaining signal on the BL1(A) fluorescence (excitation 488 nm, 530/30 Filter) recorded. To assess the amount of total harvestable CB_2_-GFP in a given volume of cell culture, viable cell density of the same sample was determined using a Vi-Cell XR cell counter and multiplied by the geometric mean of BL1 (A) fluorescence. To detect CB_2_ or CB_2_-GFP expression on the plasma membrane, 1 × 10^6^ cells were spun down, resuspended in 1 mL cold Flow Cytometry Staining Buffer (eBioscience, 00-4222-26) containing 0.25 μg of Alexa Fluor 647 conjugated anti CB_2_-antibody (R&D systems, Cat No FAB36551R) and incubated on ice for 20 min. Cells were washed twice with cold Flow Cytometry Staining Buffer before analysis on the Attune NxT. An identical strategy as described above was used to quantify CB_2_ in the plasma membrane, except that the fluorescent signal was detected on the RL1(A) channel (excitation 637 nm, 670 nm/14 nm Filter) to measure CB_2_ concentration in membranes.

### Preparation of cell membranes

Cell membranes were prepared as described earlier^5^. Briefly, cells from 30 to 40 mL of culture were collected by centrifugation, washed one time with PBS buffer, and then re-suspended in a minimal volume (5 mL) of ice-cold PBS supplemented with protease inhibitor cocktail (Roche Cat No 4693116001). The cell suspension was passed twice through a French Press at 20,000 PSI, the crude extract collected and subjected to centrifugation at 15,000×*g* for 30 min to remove cell debris. The supernatant was subjected to high-speed centrifugation (150,000×*g*, 1 h), the pellet washed once with PBS and resuspended in a minimal volume of cold PBS supplemented with 15% sucrose and a protease inhibitor cocktail. The membrane preparation was aliquoted into 1.5 mL Eppendorf tubes and snap frozen in liquid nitrogen.

### Purification of recombinant CB_2_ receptor from *E.coli* cells

Biomass containing CB_2_-130 fusion protein^3^ was homogenized in 50 mL or 100 mL batches using a Potter–Elvehjem Homogenizer in two passes, and cells disrupted in an Avestin Homogenizer. The receptor was extracted with a mixture of detergents (solubilization buffer): dodecylmaltoside (DDM, 1%, w/v), CHAPS (0.5% w/v) supplemented with cholesteryl hemisuccinate (CHS, 0.1%, w/v) and CP-55,940 (10 μM) as described previously^6^. For purification, the content of DDM was reduced to 0.1%, w/v. The addition of a high affinity ligand is essential for stabilization of the receptor throughout protein purification^36^. Fusion protein CB_2_-130 was purified by affinity chromatography on Ni-NTA Sepharose, the expression partner removed by treatment with TEV protease, and the resulting CB_2_ receptor isolated by chromatography on a 5-mL StrepTactin XT column as described earlier^3,6,34^. Resin was washed with 10 column volumes (CV) of 50 mM HEPES buffer pH 7.5 supplemented with 100 mM NaCl, 0.25 mM Façade-TEG/0.025 mM CHS and 10 μM CP-55,940 (Façade-TEG buffer), and the protein eluted by a slow flow (0.1 mL/min) of Façade-TEG buffer supplemented with 50 mM biotin. Purified CB_2_ was concentrated in a centrifugal spin concentrator (Orbital Biosciences, Topsfield, MA, USA) with a 30 kDa molecular mass cut off and washed four times with 4 mL of Façade-TEG buffer to remove biotin. The protein concentration was determined with a Bio-Rad DC kit. Glycerol was added to a final concentration of 15% (v/v), and aliquots of protein in Eppendorf tubes flash-frozen in liquid nitrogen and stored at − 80 °C.

### Purification of recombinant CB_2_ receptor from Expi293F™ cells

The biomass was collected after 48 h post-induction. CB_2_ receptor was extracted with a mixture of DDM, CHAPS and CHS and purified by two successive rounds of chromatography on Ni-NTA and StrepTactin XT affinity resins, essentially following the procedure described above for the *E.* *coli*-produced receptor. The CB_2_ protein was eluted from StrepTactin XT resin with 50 mM biotin in Façade-TEG buffer and concentrated to 2–5 mg/mL on a spin-concentrator with a 30 kDa MWCO. Glycerol was added to a final concentration of 15% (v/v), the CB_2_ protein was snap-frozen in liquid nitrogen and stored at − 80 °C.

### Protein analysis

The recombinant CB_2_ protein was detected by Western blot with mouse monoclonal antibody against human cannabinoid receptor CB_2_ (R&D Systems) or monoclonal anti-His6 antibody (Qiagen). The Western blot was developed with anti-mouse HRP antibody (1:5000 dilution) and visualized by chemiluminescence with Gel Logic imaging system (Kodak). The concentration of the detergent-solubilized protein was determined with a UV–Vis spectrometer (Agilent Technologies) using DC Protein Assay Reagent (BioRad), and bovine serum albumin (BSA) as protein standard.

### Identification of post-translational modifications in purified CB_2_ protein

Post-translational modifications in CB_2_ purified from Expi293F and Expi293F GNTI^−^ cells were analyzed by LC/MS/MS by Poochon Proteomics Solutions (Frederick, MD)**.**

### Preparation of sample for LC/MS/MS analysis for glycosylation

25 μg of protein samples were digested by trypsin either (a) without treatment or (b) upon treatment with deglycosylation Mix II [Protein Deglycosylation Mix II (P6044S, Biolabs)] according to manufacturer’s instructions. After the treatment, both a and b solutions were mixed with SDS-PAGE sample buffer, heated at 95 °C for 10 min, and separated on a 4–12% Bis–Tris gel. Upon staining with Simple Blue, the target protein bands were collected, treated with DTT followed by alkylation with iodoacetamide, and further digested by trypsin. The digested peptide mixture was then concentrated and desalted using C18 Zip-Tips (EMD Millipore). Reconstituted, desalted peptides were dissolved in 20 μL of in 0.1% formic acid and analyzed by LC/MS/MS.

### Preparation of protein samples for analysis of palmitoylation

The following CB_2_ mutants were expressed and purified from Expi293F cells and analyzed for palmitoylation: WT, C313S, C320S and C360S. Purified proteins were separated on a 4–12% Bis–Tris gel without pre-treatment with reducing agent, and the gel stained by Simple Blue. The target protein band of each sample was collected in two tubes and digested with chymotrypsin and/or trypsin without reduction by DTT and alkylation by iodoacetamide. The digested peptide mixture was then concentrated and desalted using C18 Zip-Tips. Reconstituted desalted peptides were dissolved in 20 µL of 0.1% formic acid analyzed by LC/MS/MS.

### LC/MS/MS analysis

The LC/MS/MS analysis of protein samples was carried out using a Thermo Scientific Q-Exactive hybrid Quadrupole-Orbitrap Mass Spectrometer and a Thermo Dionex UltiMate 3000 RSLCnano System. Peptide mixtures from each sample were loaded onto a peptide trap cartridge at a flow rate of 5 μL/min. The trapped peptides were eluted onto a reversed-phase PicoFrit column (New Objective, Woburn, MA, USA) using a linear gradient of acetonitrile (3–36%) in 0.1% formic acid. The elution duration was 110 min at a flow rate of 0.3 μL/min. Eluted peptides from the PicoFrit column were ionized and sprayed into the mass spectrometer, using a Nanospray Flex Ion Source ES071 (Thermo) under the following settings: spray voltage, 1.8 kV, Capillary temperature, 250 °C. Raw data files were searched against the human protein sequence database containing HPHL1 mutations using the Proteome Discoverer 1.4 software (Thermo, San Jose, CA) using the SEQUEST algorithm. Carbamidomethylation (+ 57.021 Da) of cysteines was fixed modification, and Deamidation Q/N-deamidated (+ 0.98402 Da), Oxidation/+ 15.995 Da (M), O-GalNAc/+ 203.079 Da (S, T), Acetyl/+ 42.011 Da (K), Phospho/+ 79.966 Da (S, T, Y), HexNAc/+ 203.079 Da (N), HexNAc(2)/+ 406.159 Da (N), Hex1HexNAc/+ 365.132 Da (N), and Hex(5)HexNAc(4)/+ 1622.582 Da (N) were set as dynamic modifications. The minimum peptide length was specified to be five amino acids. The precursor mass tolerance was set to 15 ppm, whereas fragment mass tolerance was set to 0.05 Da. The maximum false peptide discovery rate was specified as 0.01.

### Purification of Gα_i1_ and Gβ_1_γ_2_ subunits

Myristoylated recombinant *Gα*_*i1*_ was produced in *E.* *coli*, expressing both *Gα*_*i1*_ and *N*-myristoyltransferase, following a previously published procedure^52^.

Heterodimeric G_β1γ2_ were expressed in Sf9 cells infected with baculoviruses encoding these subunits. P2 membranes were prepared, extracted with 1% sodium cholate, and G_β1γ2_ purified essentially as described previously^53^. The purified proteins were stored in a solution of 10 mM MOPS, pH 7.5, 1 mM MgCl_2_, 100 mM NaCl with 8 mM CHAPS at − 80 °C.

### Activation of G protein in an in vitro coupled assay

Activation of G proteins by the recombinant CB_2_ was performed according to a previously reported protocol^34^. Briefly, either the cell membranes expressing CB_2_ (2 μg total protein per sample) or the purified CB_2_ (5–50 ng) in Façade-TEG/ CHS micelles were dispensed into pre-siliconized glass tubes containing 10 mM MOPS supplemented with 0.1% (w/v) BSA and 10 μM CP-55,940. Upon addition of a mixture of Gα_i1_ (100 nM) and Gβ_1_γ_2_ (500 nM), the tubes were incubated on ice for 30 min. The reaction was started by addition of (final concentrations) MOPS buffer pH 7.5 (50 mM), EDTA (1 mM), MgCl_2_ (3 mM), GDP (4 μM), BSA (0.3% w/v), NaCl (100 mM), DTT (1 mM) and an appropriate amount of^35^ S-γ-GTP, and tubes transferred rapidly to water bath set at 30 °C. The total volume of the reaction was 50 μL. Incubation continued for 20 min and was terminated by addition of 2 mL ice-cold stop solution TNMg (20 mM Tris–HCl pH 8.0, 100 mM NaCl, 25 mM MgCl_2_). The reaction was rapidly filtered through 0.45 μm nitrocellulose filters (EMD Millipore). Filters were washed with 4 × 2 mL of cold TNMg buffer, dried, placed into scintillation vials and counted upon addition of ScintiSafe Econo F scintillation liquid (Fisher).

### Analysis of thermostability

Thermostability of CB_2_was determined according to protocols published previously^34^. Thermostability in membranes was tested after re-suspension, on ice of membrane preparations expressing CB_2_ in a reaction buffer containing 10 mM MOPS pH 7.0 and 10 μM CP-55,940. The concentration of membrane proteins in reaction was 0.1–0.2 mg/mL. The 30 μL aliquots were subjected to a temperature gradient of 1 °C/min, withdrawn at time intervals, and placed on ice prior to the G protein activation analysis.

### Enzymatic deglycosylation

Enzymatic deglycosylation of the purified receptor with PNGase F (New England Biolabs) was performed as follows. An amount of 50 μg of purified receptor in 0.25 mM Façade-TEG/0.025 mM CHS/10 μM CP-55,940 were mixed with 10 μg of PNGase F in a total volume of 20 μL and incubated for 4 h at 10 °C. The control samples of CB_2_ protein were incubated at the same conditions but without the PNGase F. Upon completion of the reaction, 3 μL aliquots were withdrawn and analyzed by SDS-PAGE and Western blot to confirm the successful deglycosylation. The remainder of the sample was diluted 200-fold with Façade-TEG buffer and subjected to temperature treatment as indicated in the text.

### NMR analysis

Composition of NMR samples was analyzed by solubilization of 10–30 μL of protein solubilized in detergent, followed by acquisition of ^1^H NMR spectra on an AV800 spectrometer (Bruker Biospin Inc.). The ^1^H-^13^C HSQC spectra of solubilized protein were recorded on an AV600 spectrometer equipped with a cryoprobe (Bruker BiopSpin, Inc.).

## Supplementary information

Supplementary Information
