# Supplementary material for: Thermostability of a recombinant G protein-coupled receptor expressed at high level in mammalian cell culture
Source: Sci Rep. 2020 Oct 8;10:16805. doi: 10.1038/s41598-020-73813-7 (PMC7546613; doi:10.1038/s41598-020-73813-7)
Supplement: Supplementary file 1 — Supplementary Information [file 41598_2020_73813_MOESM1_ESM.pdf]

Thermostability of a recombinant G protein-coupled receptor expressed at high level in mammalian cell cultures

Alexei Yeliseev[1]*, Arjen van den Berg[2], Lioudmila Zoubak[1], Kirk Hines[1], Sam Stepnowski[2], Kyle Williston[2], Wanhua Yan[2], Klaus Gawrisch[1], Jonathan Zmuda[2]

[1] National Institute on Alcoholism and Alcohol Abuse, NIH, 5625 Fishers Lane, Room 3N11, Rockville, MD 20815, USA and

[2] ThermoFisher Scientific, 7335 Executive Way, Fredrick, MD 21704, USA

*Address correspondence to:
Alexei Yeliseev
National Institute on Alcoholism and Alcohol Abuse, NIH,
5625 Fishers Lane, Room 3N11, Rockville, MD 20815, USA
Phone: +1 3014430552
Email: yeliseeva@mail.nih.gov

Supplementary Data

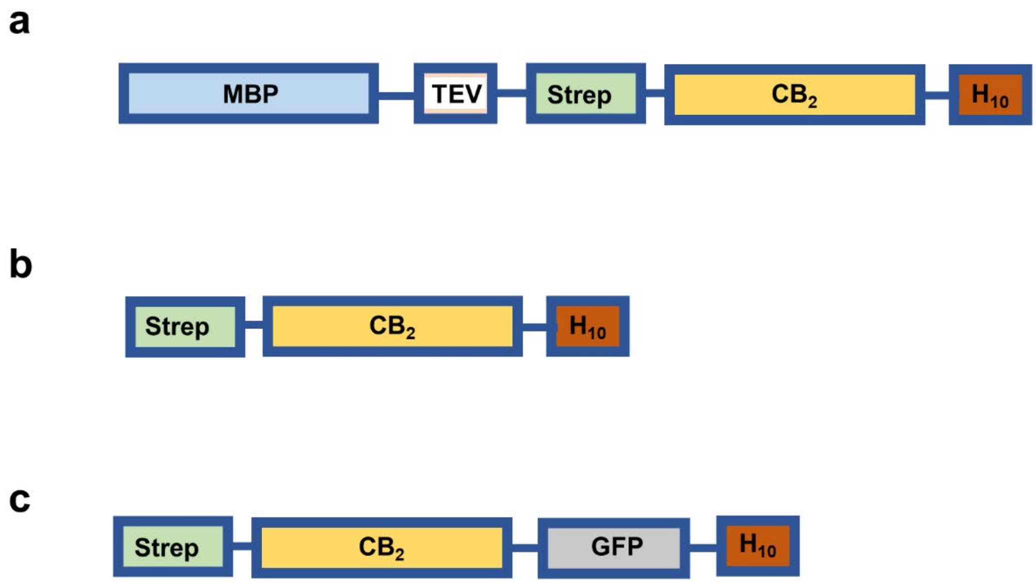

Supplementary Figure 1. **Constructs for expression of CB_2**. **a**, MBP-CB_2 fusion construct for expression in *E. coli* BL21(DE3).; **b**, CB_2 construct for expression in mammalian cell lines, in pCDNA3.4; **c**, CB_2-GFP construct for expression in Expi cell lines, in pCDNA3.4.

Expi293F™

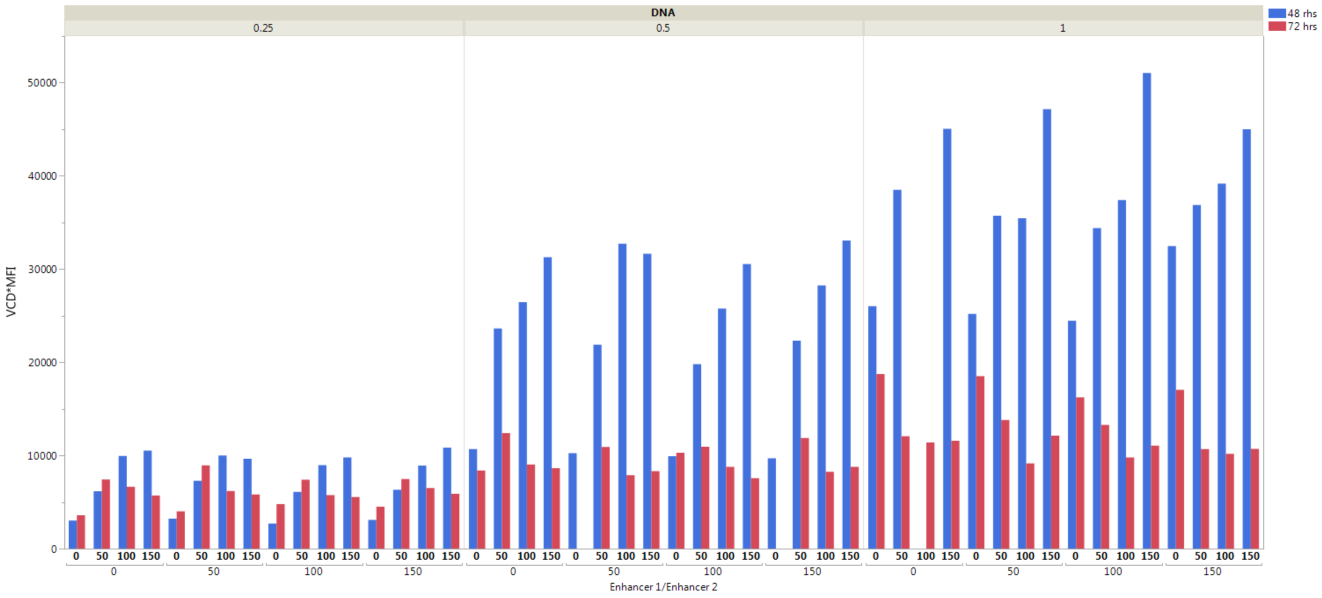

Supplementary Figure 2. DOE of CB_2 expression in Expi293F™. The Expi293F™ expression system was investigated for expression of CB_2-GFP by FACS. Three concentrations of DNA (top label) were combined with 4 amounts of enhancers 1 (x-axis upper bold label) and 2 (x-axis lower label). Data are presented as geometric mean of GFP MFI multiplied by VCD as determined by Vi-Cell XR to represent total harvestable CB_2 per volume of culture. For simplicity, only data from 48hrs (blue bars) and 72 hrs (red bars) are shown as signal clearly peaked at 48 hrs. Data presented are from a single experiment with single samples.

Expi293F™ GNTI-

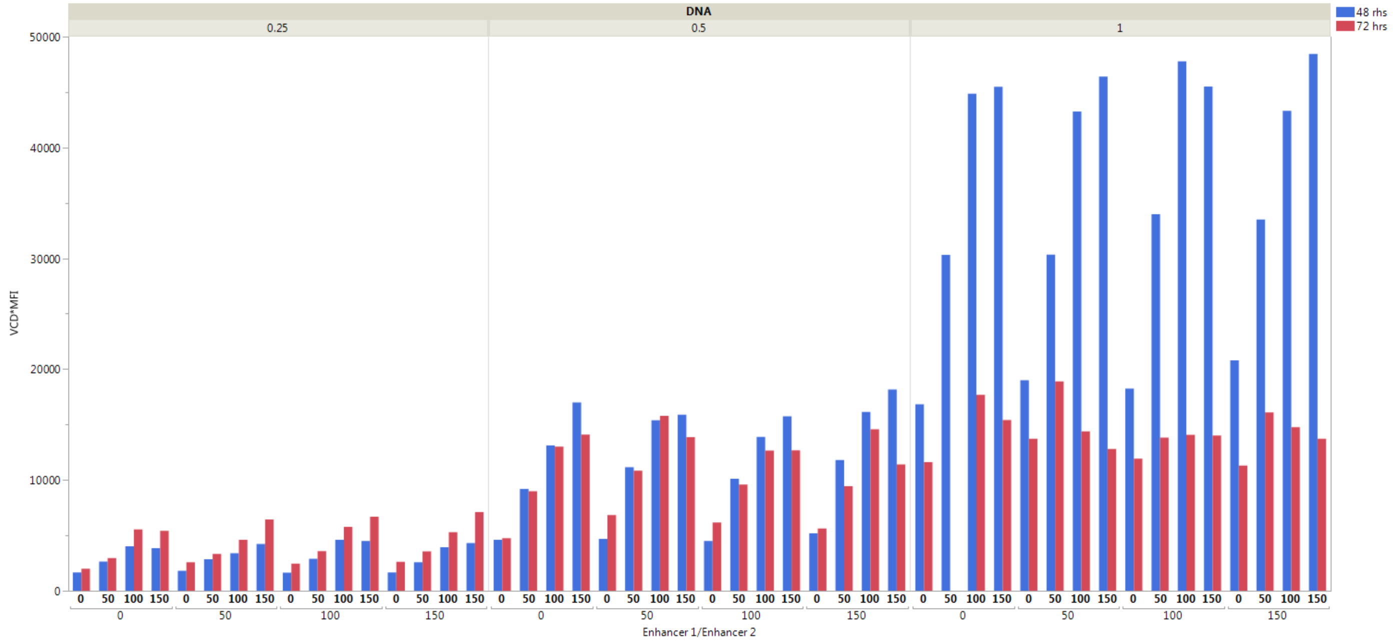

Supplementary Figure 3. DOE of CB_2 expression in Expi293F™ GNTI$^-$. The Expi293F™ GNTI$^-$ expression system was investigated for expression of CB_2-GFP by FACS. Three concentrations of DNA (top label) were combined with 4 amounts of enhancers 1 (x-axis upper bold label) and 2 (x-axis lower label). Data are presented as geometric mean of GFP MFI multiplied by VCD as determined by Vi-Cell XR to represent total harvestable CB_2 per volume of culture. For simplicity, only data from 48hrs (blue bars) and 72 hrs (red bars) are shown as signal clearly peaked at 48 hrs. Data presented are from a single experiment with single samples.

a

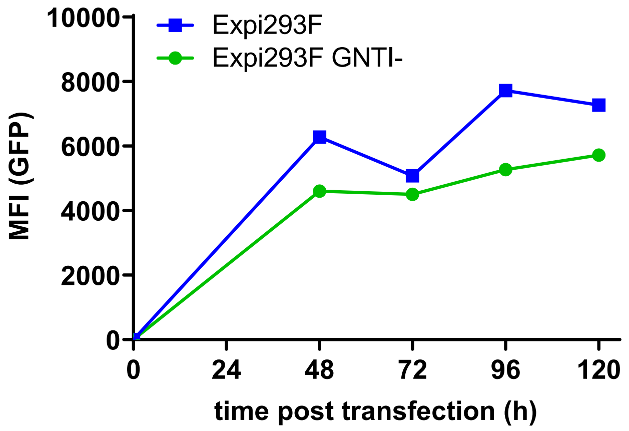

b

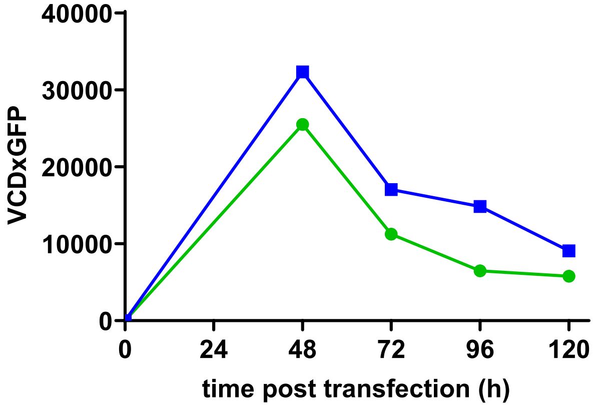

Supplementary Figure 4. Effect of addition of stabilizing ligand CP-55,940 on CB_2 expression. The mammalian cell systems were transfected using optimized conditions determined by the DOE shown in figure S2 and S3. Single cell GFP intensity is depicted in **a**, whereas **b** depicts the effect of multiplication of GFP MFI by VCD. Data presented are from a single experiment with single samples

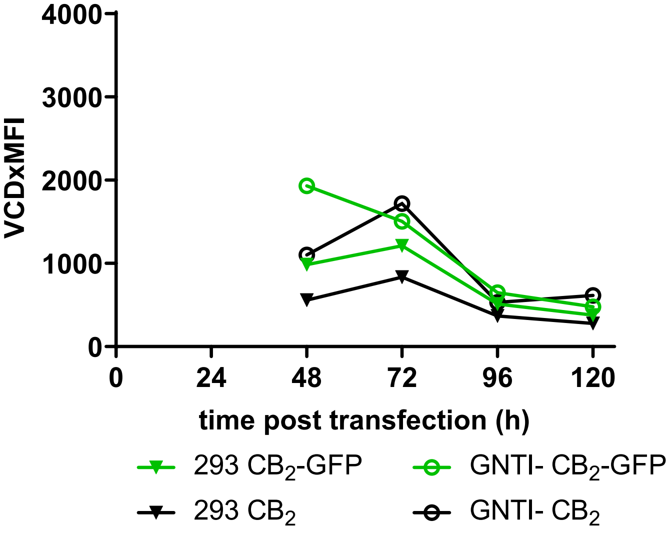

Supplementary Figure 5. Comparison of membrane expression of GFP-tagged CB_2 vs untagged CB_2. To assess whether CB_2 expressed as a GFP fusion protein followed similar behavior as untagged CB_2, Expi293F™ and Expi293F™ GNTI$^-$ cells were transfected with the 2 respective constructs and expression at the cell membrane was measured by FACS using an antibody raised against the extracellular domain of CB_2. Green lines represent GFP-fused CB_2, black lines the untagged CB_2. Data presented are from a single experiment with single samples.

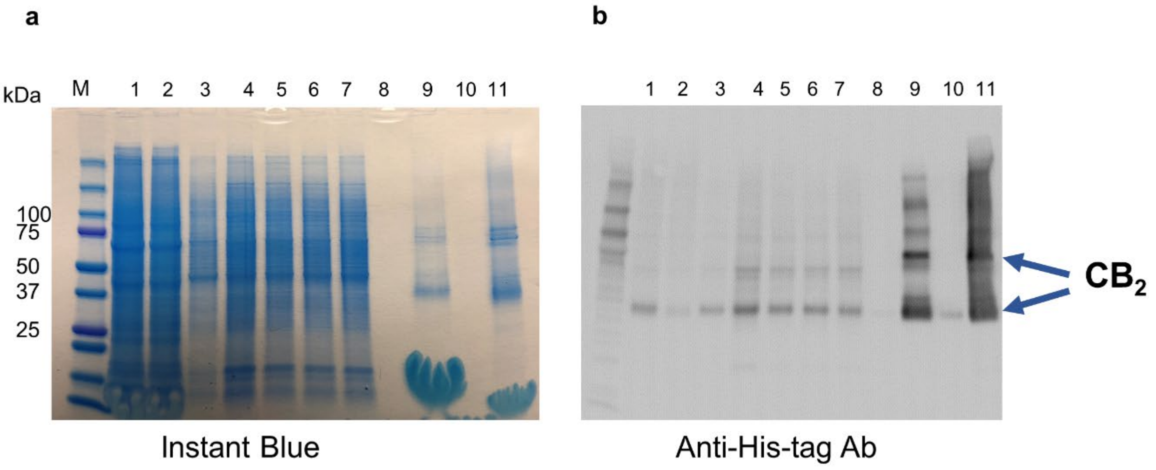

Supplementary Figure 6. Purification of CB_2 from Expi293F^{TM} GNTI^{-} cells. **a**, Instant Blue-stained SDS-PAGE, 10%; **b**, Western blot probed with anti-Histag antibody. CB_2 was purified as described in Methods. Lane1, solubilized total membrane proteins (crude extract); lanes 2-7 elution fractions from Ni-NTA and StrepTactin XT columns; 8, 10- wash of the StrepTactin column; 9, 11 – fractions of purified CB2. Arrows indicate the position of CB_2 monomer and dimer. Higher immunoreactive bands correspond to the oligomeric forms of CB_2 that are formed during SDS-PAGE separation.

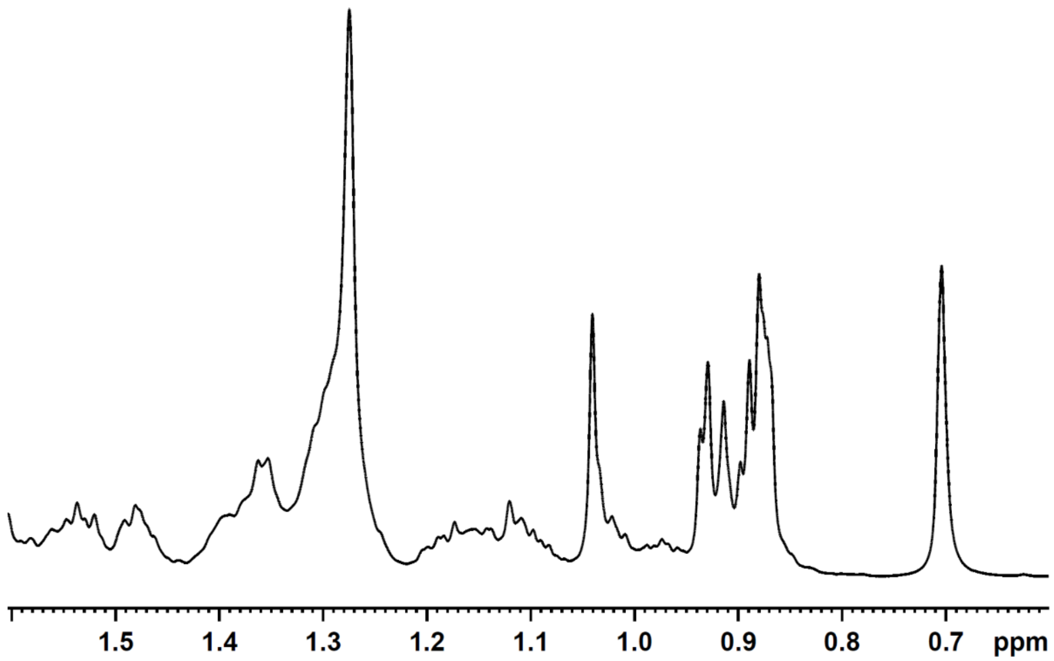

Supplementary Figure 7. Determination of detergent- and lipid composition of CB_2 micelles. ^{1}H NMR spectrum of 10µL of the CB_2 in Façade-TEG/phospholipid/CHS micelles, dissolved in deuterated chloroform/ methanol (1/1, vol/vol), recorded at 22ºC. Area of resonances at 0.7ppm (Façade-TEG + CHS), 1.04 ppm (CHS) and 1.28 ppm (phospholipid) were used to determine concentrations in the original sample: CB_2: 74 µM, Façade-TEG: 1.09 mM, phospholipid: 83 µM, CHS: 357 µM.

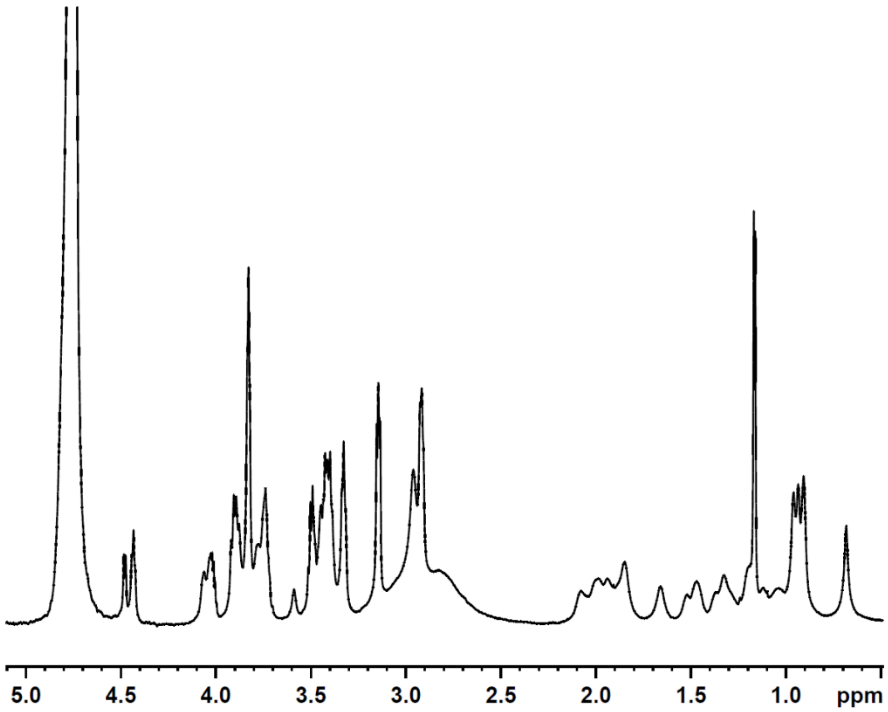

Supplementary Figure 8. Determination of diffusion rates of Façade-TEG micelles. ^{1}H NMR spectrum of Façade-TEG (250 μM) in D_2O recorded at 22ºC. The methyl group resonance at 0.7 ppm was selected for measurement of diffusion rates of Façade-TEG molecules.

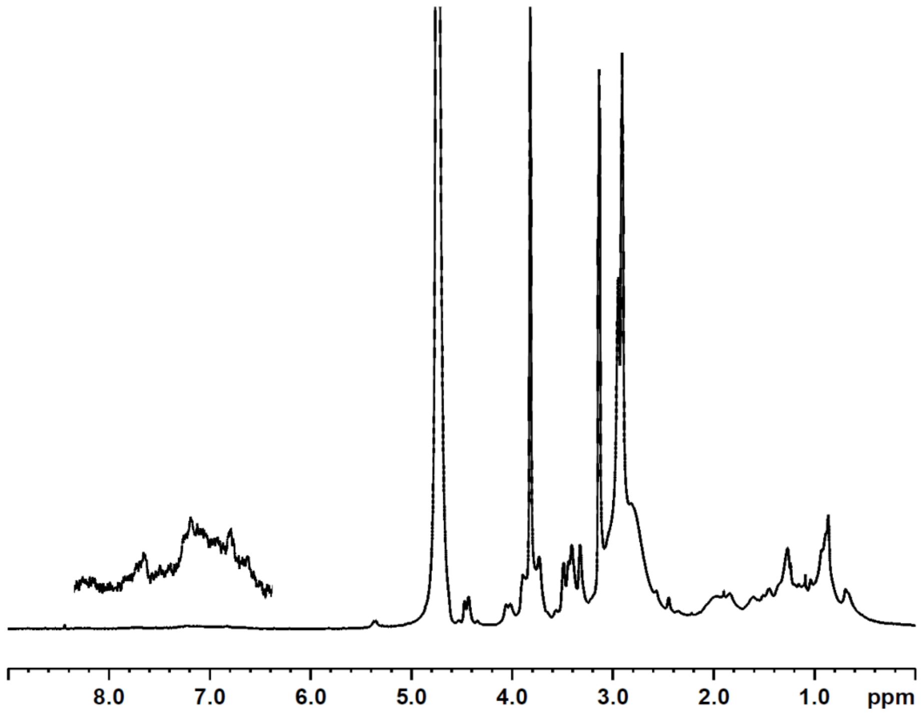

Supplementary Figure 9. Determination of diffusion rates of CB_2/Façade-TEG/CHS/phospholipids micelles. ^{1}H NMR spectrum of CB_2 solubilized in Façade-TEG/phospholipid/CHS micelles in deuterated Tris/D_2O buffer, pD 7.8, recorded at 22°C. The band of amide resonances of CB_2 from 6.8-7.6 ppm was used to measure diffusion rates of CB_2-containing Façade-TEG micelles.

a

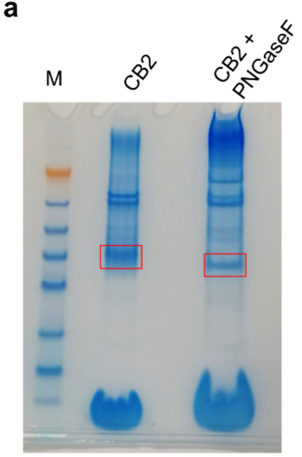

b

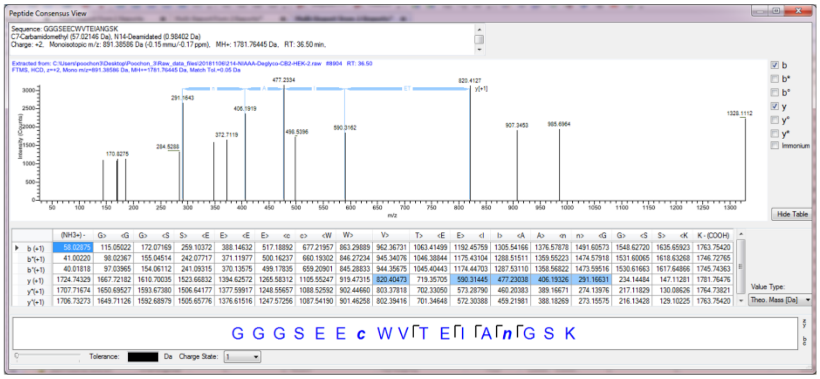

c

Result of analysis of CB2 band without deglycosylation treatment:

MWSHPQFEKG GGSGGGSGGG SAWSHPQFEK GGGSEECWVT EIANGSK **DGL DSNPMKDYMI LSGPQK** TAVA VLCTLLGLLS ALENVAVLYL ILSSHQLRRK

PSYLFIGSLA GADFLASVVF ACSFVNFHVF HGVDSKAVFL LKIGSVTMTF TASVGSLLLT AIDRYLCLR **Y PPSYKALLTR** GRALVTLGIM WVLSALVSYL

PLMGWTCCPR PCSELFPLIP NDYLLSWLLF IAFLFSGIIY TYGHVLWKAH QHVASLSGHQ DRQVPGMARM RLDVRLAKTL GLVLAVLLIC WFPVLALMAH

P PP

SLATTLSDQV KKAFAFCSML CLINSMVNPV IYALRSGEIR **SSAHHCLAHW K** KCVR **GLG SE AKEEAPR SSV TETEADGKIT PWPDSR** DLDL SDCGTHHHHH

HHHHH

Result of analysis of CB2 band with deglycosylation treatment:

Deamidated

MWSHPQFEKG GGSGGGSGGG SAWSHPQFEK **GGGSEECWVT EIA N GSKDGL DSNPMKDYMI LSGPQK** TAVA VLCTLLGLLS ALENVAVLYL ILSSHQLRRK

PSYLFIGSLA GADFLASVVF ACSFVNFHVF HGVDSKAVFL LKIGSVTMTF TASVGSLLLT AIDRYLCLR **Y PPSYK** ALLTR GRALVTLGIM WVLSALVSYL

PLMGWTCCPR PCSELFPLIP NDYLLSWLLF IAFLFSGIIY TYGHVLWK **AH QHVASLSGHQ DRQVPGMAR** M RLDVRLAKTL GLVLAVLLIC WFPVLALMAH

P P P

SLATTLSDQV KKAFAFCSML CLINSMVNPV IYALRSGEIR SS AHHCLAHW KKCVR **GLG SE AKEEAPRS SV TETEADGKIT PWPDSR** DLDL SDCGTHHHHH

HHHHH

Supplementary Figure 10. **Determination of glycosylation site by LC/MS/MS analysis of CB_2 purified from Expi293F^{TM} GNTI$^-$ cells. a**, Preparation of protein sample for LC/MS/MS. Samples were treated with Deglycosylation Mix II, separated on a 4-12% NuPAGE gel, and stained with SimplyBlue. The target protein gel-bands (red-squared) were excised for in-gel trypsin digest; **b**, LC/MS/MS analysis of tryptic digest of deglycosylated sample; **c**, Peptides detected by LC/MS/MS. Green colored sequence was detected from analysis of non-deglycosylated CB_2 band (upper panel) and deglycosylated CB_2 band (lower panel) after trypsin digest. Note: P indicates that S or T is phosphorylated; N11 is deamidated in the deglycosylated CB_2.

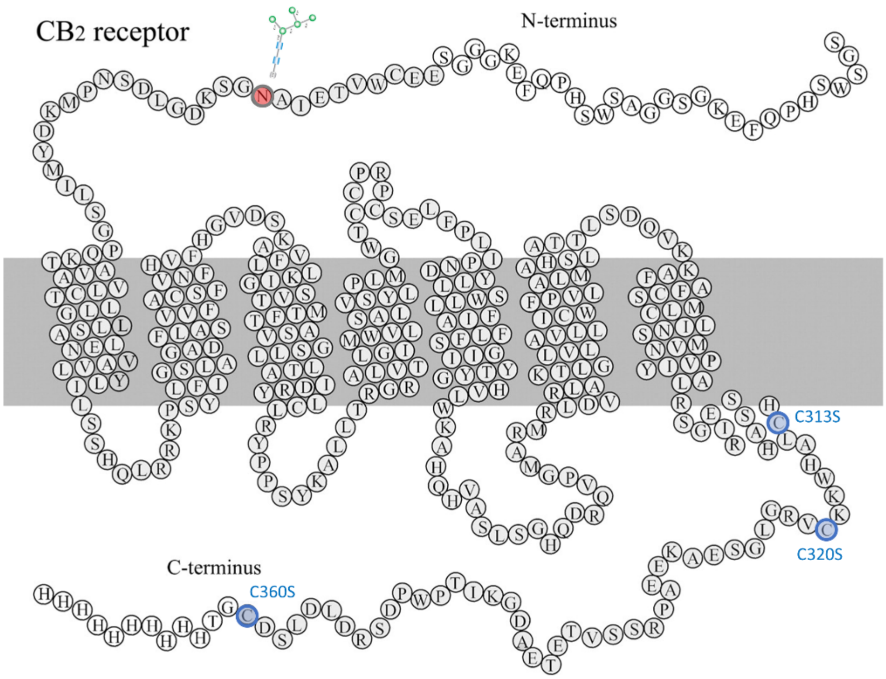

Supplementary Figure 11. **Schematic structure of the recombinant human CB_2 protein expressed in HEK cells.** Highlighted residues indicate possible sites for glycosylation and acylation.

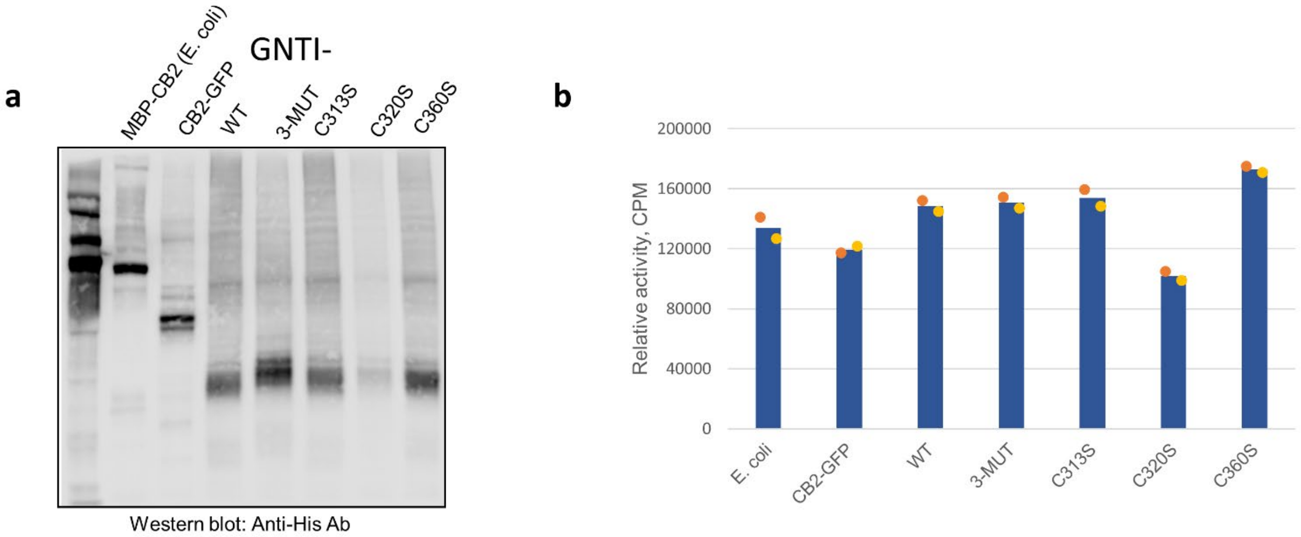

Supplementary Figure 12. **Expression of cysteine-replacement variants of CB_2 in Expi293F^{TM} GNTI$^-$ cells. a**, Western blot analysis of CB_2 levels in membrane preparations from GNTI- cells. 20 µg of membrane protein per lane. For comparison, MBP-CB_2 fusion expressed in *E. coli* cell membranes and CB_2-GFP fusion protein expressed in GNTI- cell membranes are shown. **b**, activity of CB_2 and its cysteine replacement variants as determined by G protein activation assay. 2 µg of membrane preparation per reaction performed in the presence of 5 µM CP-55,940. Results are an average of two independent measurement with individual data points shown.

a

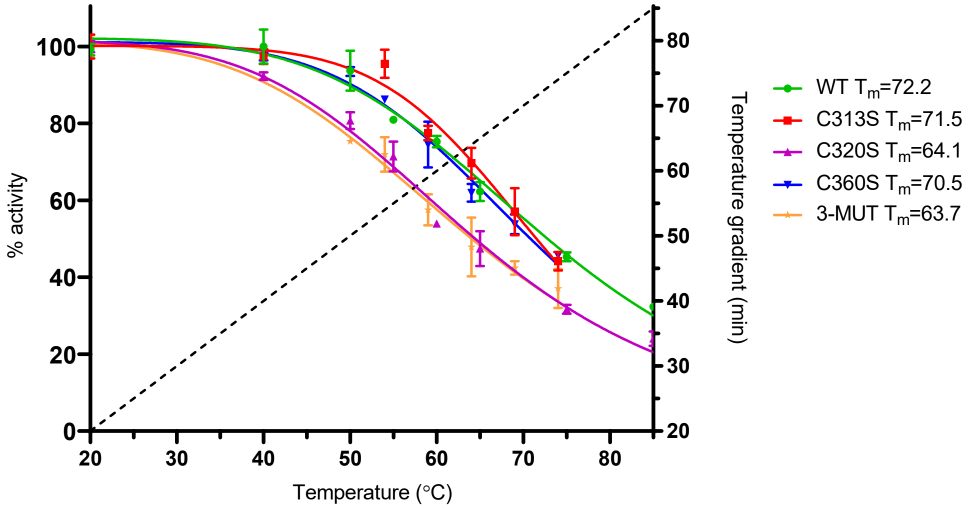

b

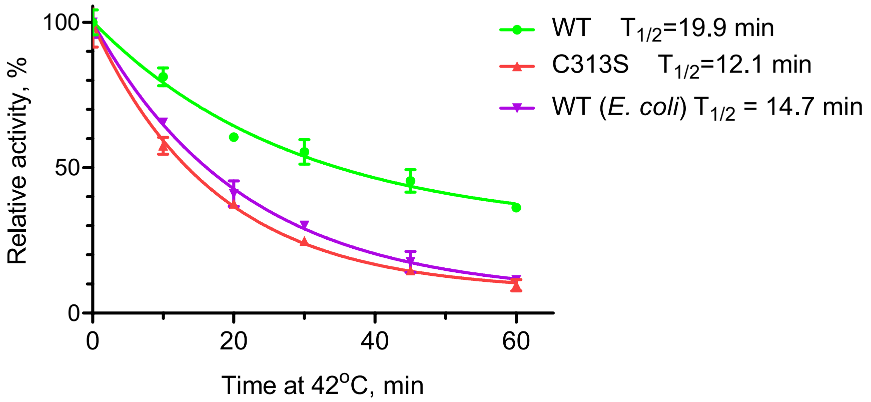

Supplementary Figure 13. **Stability of cysteine-replacement variants of CB_2 expressed in ExpiF^{TM} GNTI$^-$ cells. a**, thermoinactivation of CB_2 and variant constructs in membranes subjected to a temperature gradient of 1°C/ min; **b**, isothermal stability of purified CB_2 WT and C313S constructs in Façade-TEG/ CHS micelles at 42°C. Purified CB_2 from *E. coli* is shown for comparison. Activity of CB_2 in samples subjected to temperature treatment was determined by G protein activation test in the presence of saturating concentrations of agonist CP-55,940. Bars represent an average of two independent measurements and individual measurements are represented by dots.

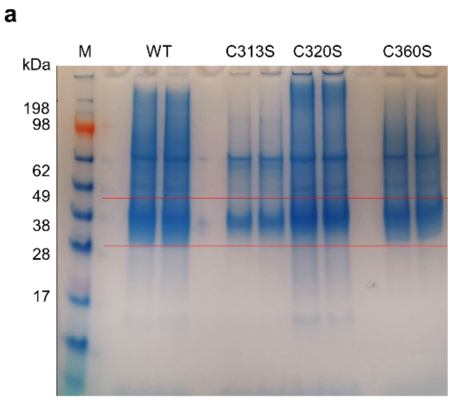

b

Site	WT	C313S	C320S	C360S
	palmitoylation			
C4	ND	ND	ND	ND
C40	ND	ND	ND	ND
C134	ND	ND	ND	ND
C284	ND	ND	ND	ND
C313	partial	ND	ND	partial
C320	ND	ND	ND	ND

Note: ND=not detected

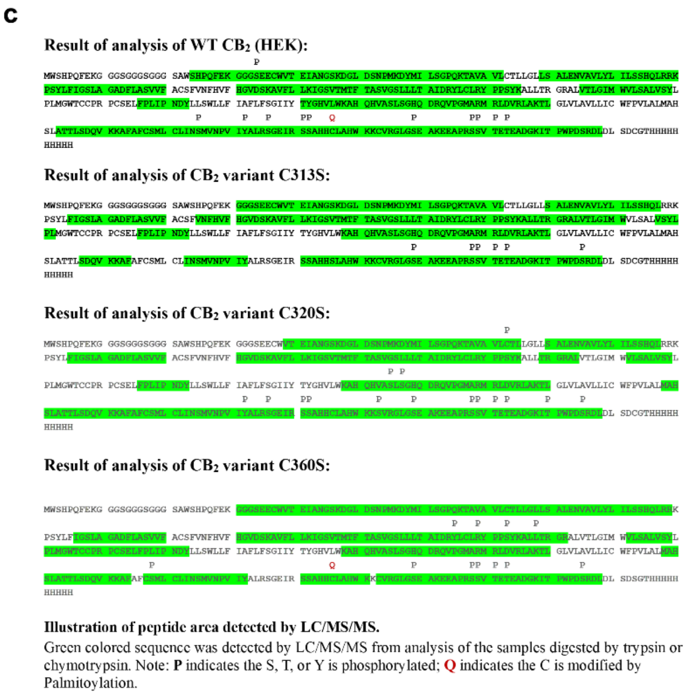

Supplementary Figure 15. **Determination of palmitoylation sites by LC/MS/MS analysis of CB_2 purified from Expi293F^{TM} GNTI$^-$ cells. a**, Preparation of protein sample for LC/MS/MS. Samples were separated on a 4-12% NuPAGE gel and stained with SimplyBlue. The target protein gel-bands (red-squared) were excised for in-gel trypsin digest; **b**, partial summary table; **c**, Peptides detected by LC/MS/MS. Green colored sequence was detected from analysis of CB_2. bands after trypsin digest. Note: P indicates the S or T is phosphorylated; Q indicates the cysteine residue modified by palmitoylation.

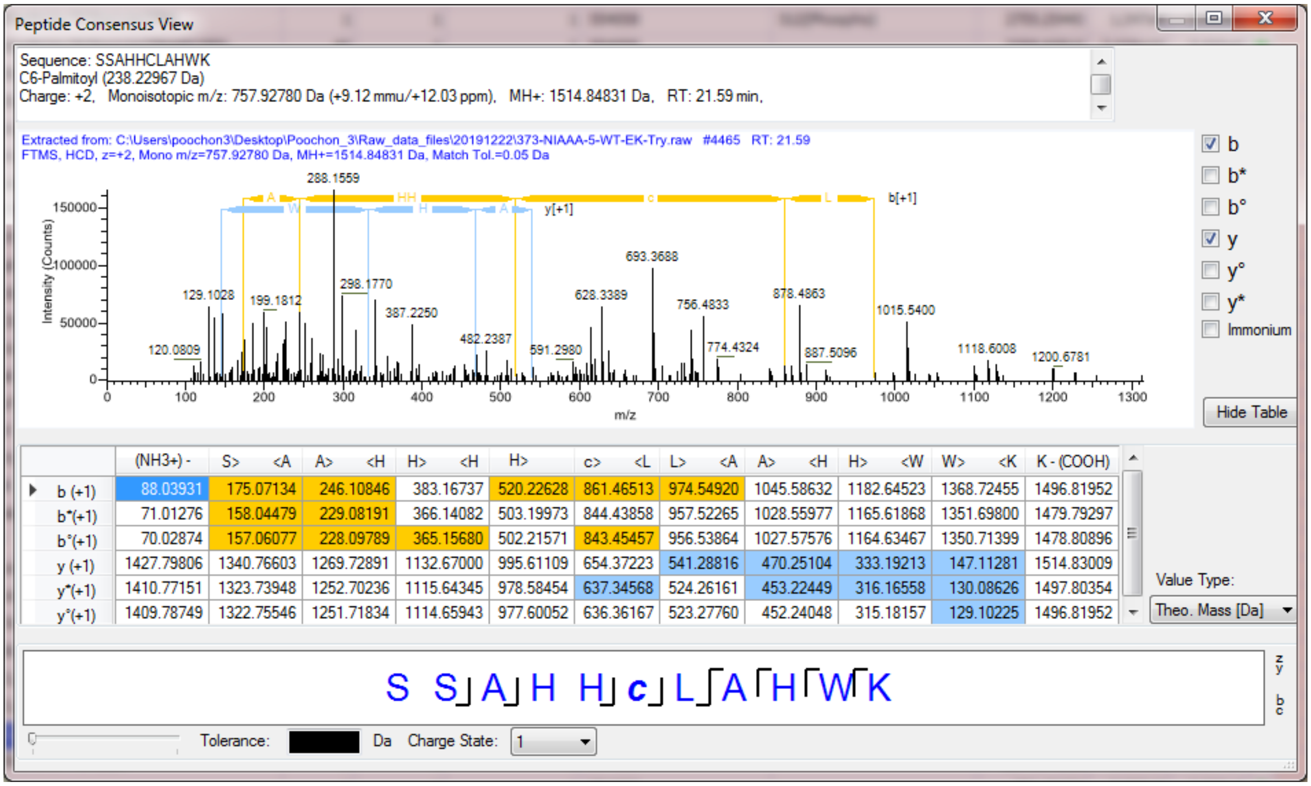

	(NH3+) -	S> <A	A> <H	H> <H	H>	c> <L	L> <A	A> <H	H> <W	W> <K	K - (COOH)
b (+1)	88.03931	175.07134	246.10846	383.16737	520.22628	861.46513	974.54920	1045.58632	1182.64523	1368.72455	1496.81952
b*(+1)	71.01276	158.04479	229.08191	366.14082	503.19973	844.43858	957.52265	1028.55977	1165.61868	1351.69800	1479.79297
b°(+1)	70.02874	157.06077	228.09789	365.15680	502.21571	843.45457	956.53864	1027.57576	1164.63467	1350.71399	1478.80896
y (+1)	1427.79806	1340.76603	1269.72891	1132.67000	995.61109	654.37223	541.28816	470.25104	333.19213	147.11281	1514.83009
y*(+1)	1410.77151	1323.73948	1252.70236	1115.64345	978.58454	637.34568	524.26161	453.22449	316.16558	130.08626	1497.80354
y°(+1)	1409.78749	1322.75546	1251.71834	1114.65943	977.60052	636.36167	523.27760	452.24048	315.18157	129.10225	1496.81952

Sequence: SSAHHCLAHWK, **C6-Palmitoyl** (238.22967 Da)

Charge: +2, Monoisotopic m/z: 757.92780 Da (+9.12 mmu/+12.03 ppm), MH+: 1514.84831 Da, RT: 21.59 min,

Identified with: Sequest HT (v1.3); XCorr:1.06, Ions matched by search engine: 0/0

Fragment match tolerance used for search: 0.05 Da; Fragments used for search: b; b-H_2O; y; y-H_2O; y-NH_3

Protein references (1): - CNR2_HUMAN Cannabinoid receptor 2, CB2-WT [Homo sapiens]

Supplementary Figure 16. Detection of palmitoylation at Cys313 by LC/MS/MS analysis of WT CB2 purified from Expi293F™ GNTI- cells. Samples were prepared and analyzed as described in Methods.

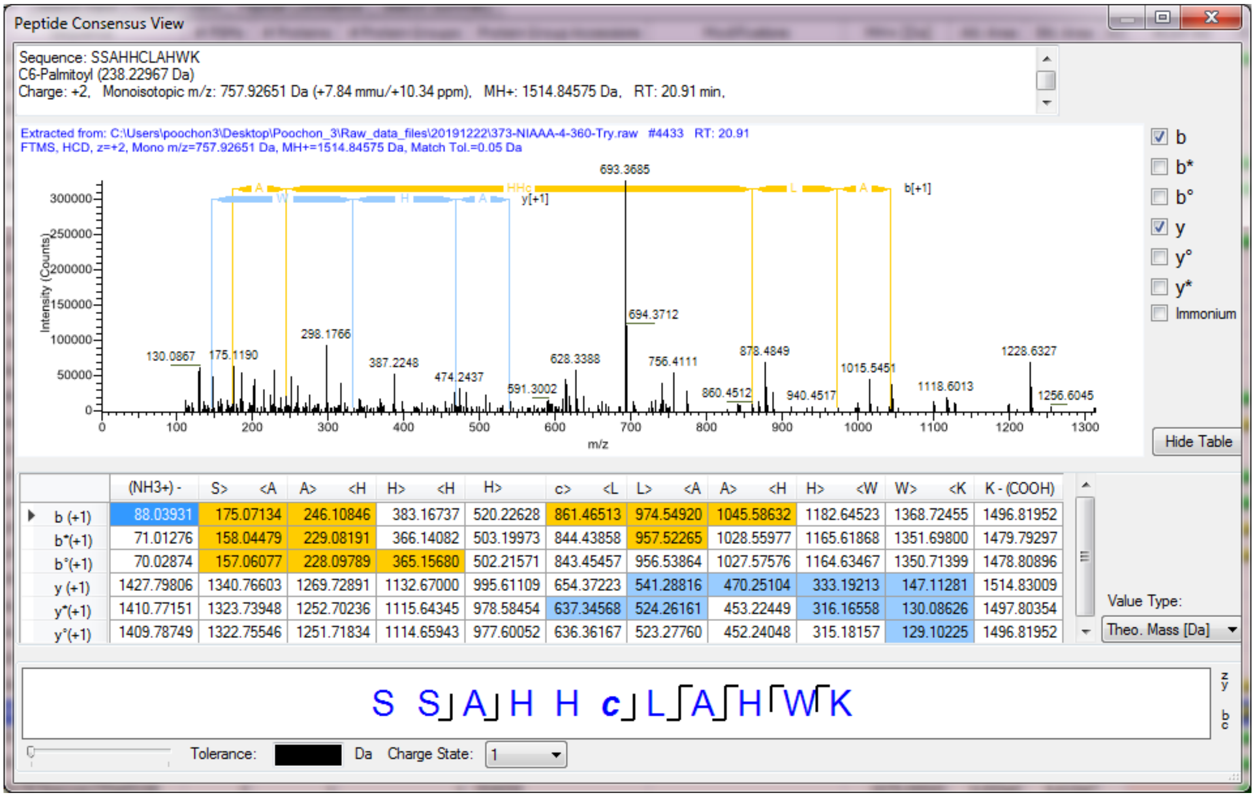

Sequence: SSAHHCLAHWK, C6-Palmitoyl (238.22967 Da)

Charge: +2, Monoisotopic m/z: 757.92651 Da (+7.84 mmu/+10.34 ppm), MH+: 1514.84575 Da, RT: 20.91 min,

Identified with: Sequest HT (v1.3); XCorr:0.88, Ions matched by search engine: 0/0

Fragment match tolerance used for search: 0.05 Da; Fragments used for search: b; b-H_2O; y; y-H_2O; y-NH_3

Protein references (1): - CNR2_HUMAN Cannabinoid receptor 2, CB2-C360S [Homo sapiens]

Supplementary Figure 17. Detection of palmitoylation at Cys313 by LC/MS/MS analysis of CB_2 C360S purified from Expi293F^{TM} GNTI$^-$ cells. Samples were prepared and analyzed as described in Methods.

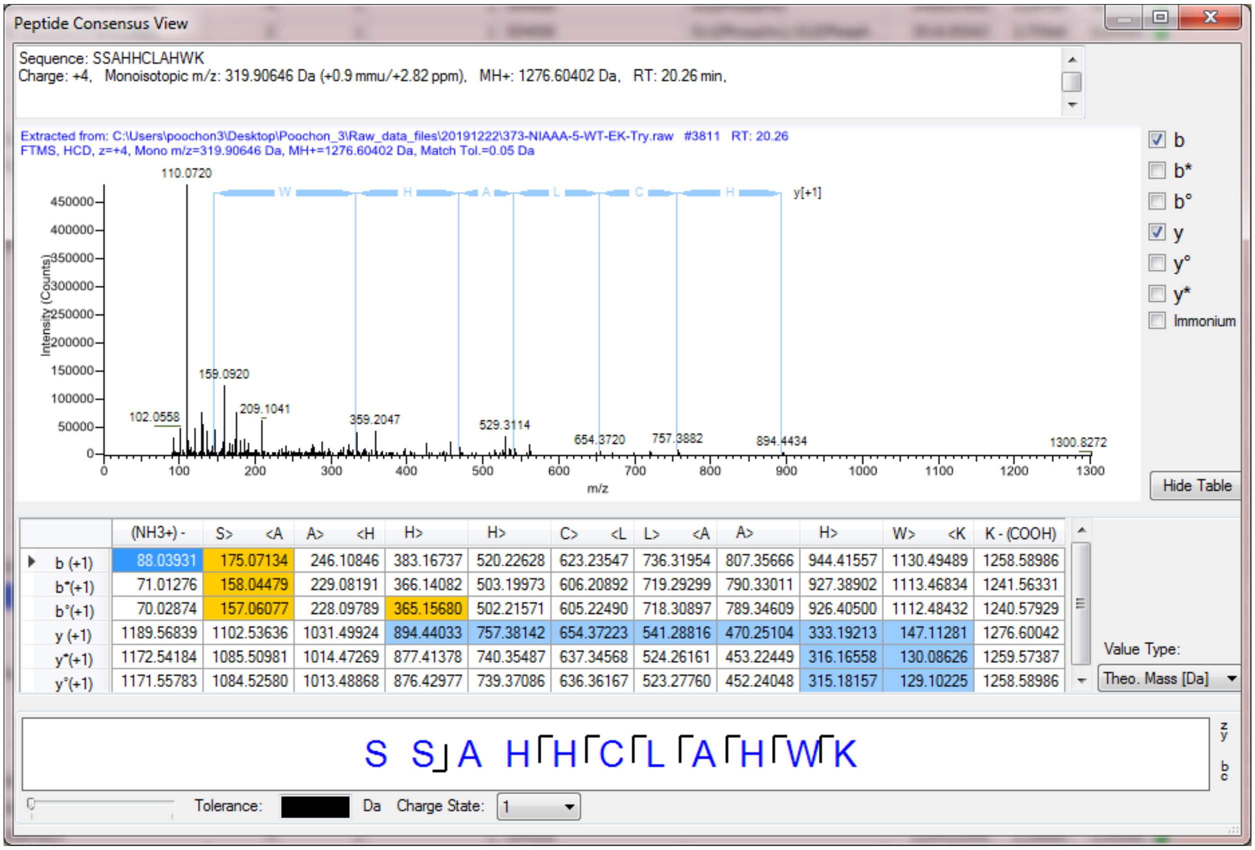

Sequence: SSAHHCLAHWK, Charge: +4, Monoisotopic m/z: 319.90646 Da (+0.9 mmu/+2.82 ppm), MH+: 1276.60402 Da, RT: 20.26 min,

Identified with: Sequest HT (v1.3); XCorr:3.12, Ions matched by search engine: 0/0

Fragment match tolerance used for search: 0.05 Da; Fragments used for search: b; b-H_2O; y; y-H_2O; y-NH_3

Protein references (1): - CNR2_HUMAN Cannabinoid receptor 2, CB2-WT [Homo sapiens]

Supplementary Figure 18. LC/MS/MS analysis of non-palmitoylated WT CB_2 purified from Expi293F^{TM} GNTI$^-$ cells. Samples were prepared and analyzed as described in Methods.

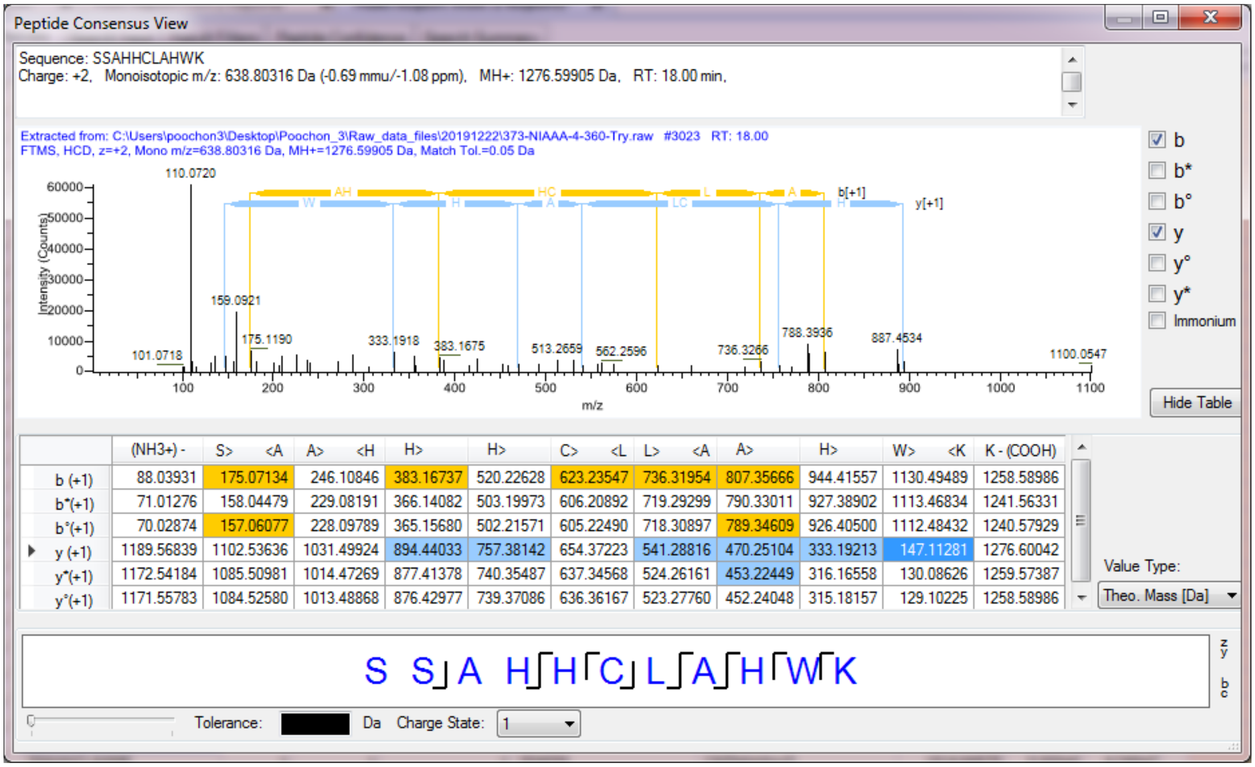

Sequence: SSAHHCLAHWK, Charge: +2, Monoisotopic m/z: 638.80316 Da (-0.69 mmu/-1.08 ppm), MH+: 1276.59905 Da, RT: 18.00 min,

Identified with: Sequest HT (v1.3); XCorr:1.94, Ions matched by search engine: 0/0

Fragment match tolerance used for search: 0.05 Da; Fragments used for search: b; b-H_2O; y; y-H_2O; y-NH_3

Protein references (1): - CNR2_HUMAN Cannabinoid receptor 2, CB2-C360S [Homo sapiens]

Supplementary Figure 19. LC/MS/MS analysis of non-palmitoylated CB_2 C360S purified from Expi293F^{TM} GNTI$^-$ cells. Samples were prepared and analyzed as described in Methods.

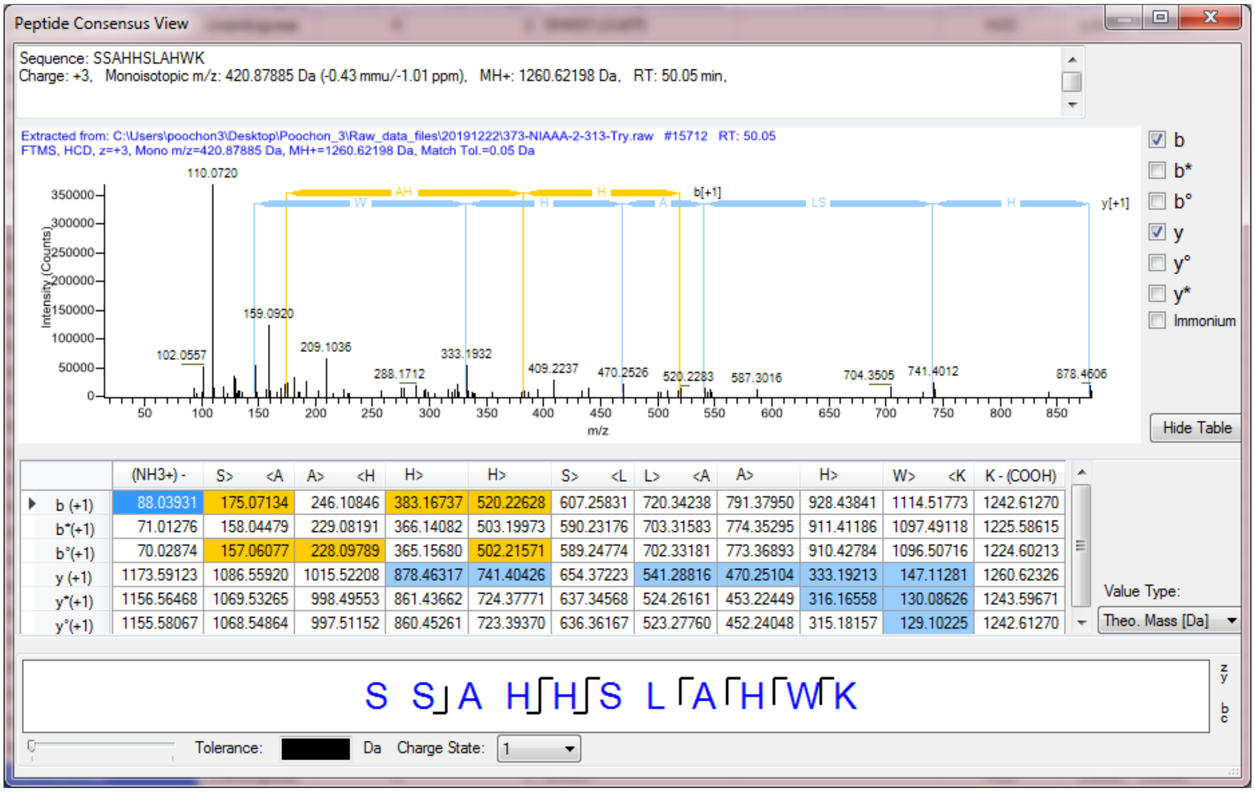

Sequence: SSAHHSLAHWK, Charge: +3, Monoisotopic m/z: 420.87885 Da (-0.43 mmu/-1.01 ppm), MH+: 1260.62198 Da, RT: 50.05 min,

Identified with: Sequest HT (v1.3); XCorr:3.14, Ions matched by search engine: 0/0

Fragment match tolerance used for search: 0.05 Da; Fragments used for search: b; b-H_2O; y; y-H_2O; y-NH_3

Protein references (1): - CNR2_HUMAN Cannabinoid receptor 2 C313S, CB2-C313S [Homo sapiens]

Supplementary Figure 20. **LC/MS/MS analysis of non-palmitoylated CB_2 C313S purified from Expi293F^{TM} GNTI$^-$ cells.** Samples were prepared and analyzed as described in Methods.

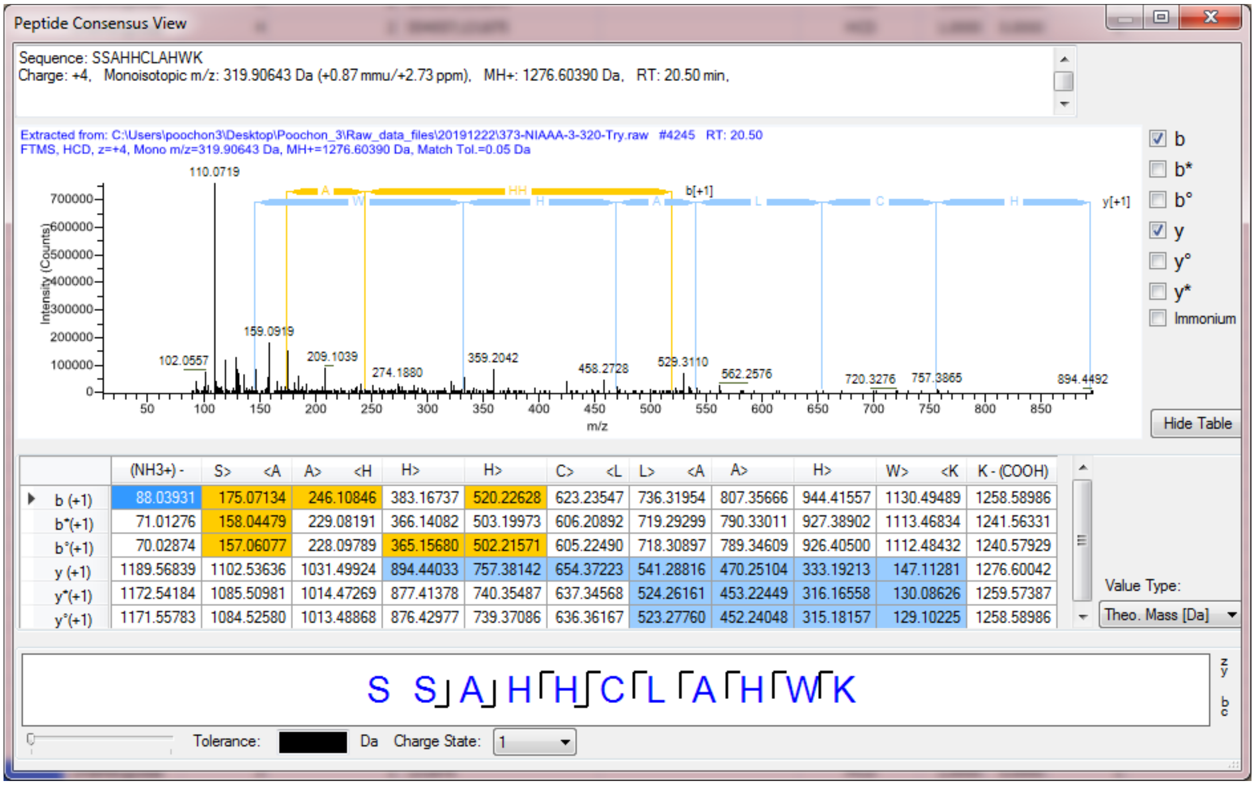

Sequence: SSAHHCLAHWK, Charge: +4, Monoisotopic m/z: 319.90643 Da (+0.87 mmu/+2.73 ppm), MH+: 1276.60390 Da, RT: 20.50 min,

Identified with: Sequest HT (v1.3); XCorr:3.24, Ions matched by search engine: 0/0

Fragment match tolerance used for search: 0.05 Da; Fragments used for search: b; b-H_2O; y; y-H_2O; y-NH_3

Protein references (3): - CNR2_HUMAN Cannabinoid receptor 2-C320S, CB2-C320S [Homo sapiens]

Supplementary Figure 21. LC/MS/MS analysis of non-palmitoylated CB_2 C320S purified from Expi293F^{TM} GNTI$^-$ cells. Samples were prepared and analyzed as described in Methods.

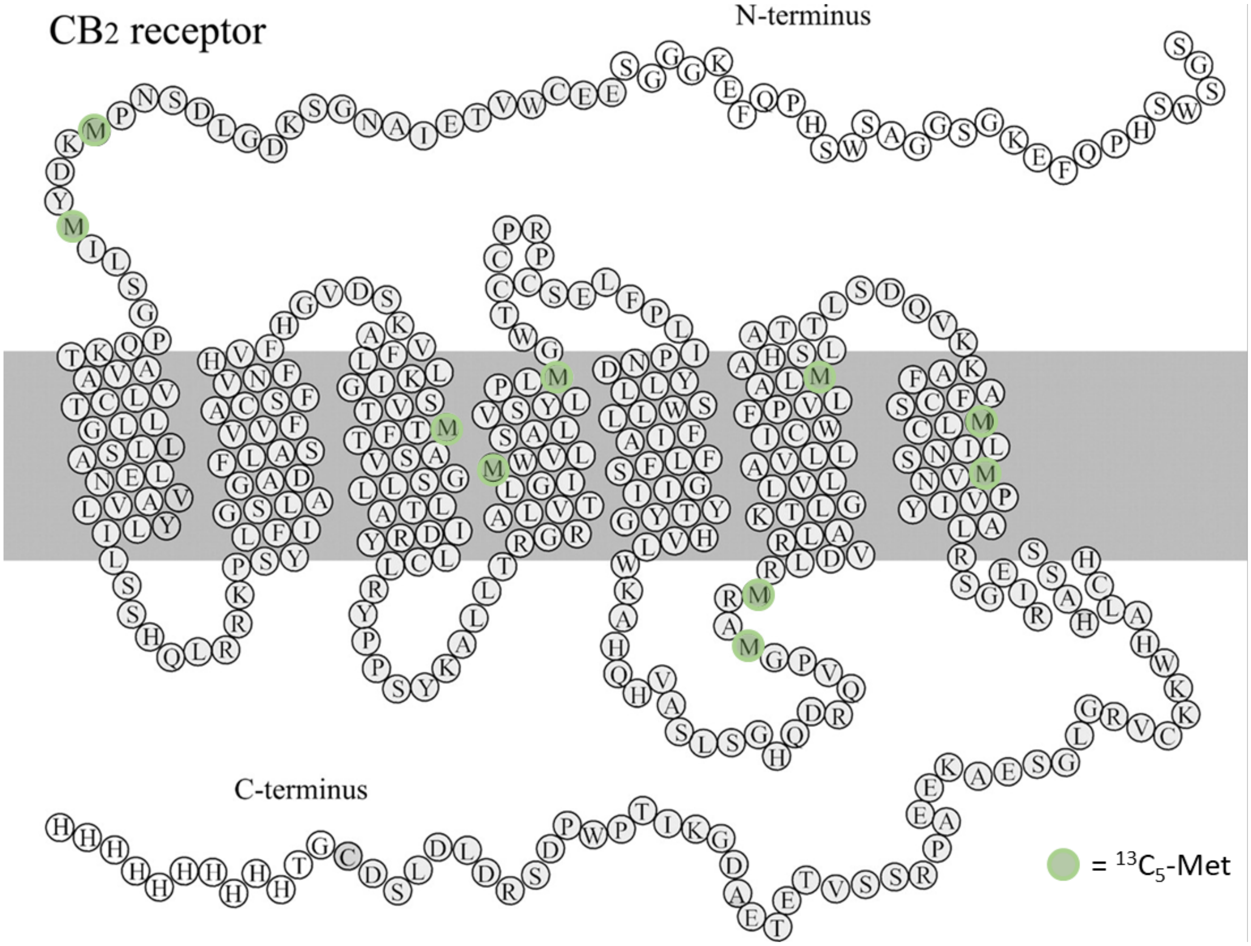

Supplementary Figure 22. Schematic structure of the recombinant CB_2 labeled with $^{13}C_5$-methionine. Highlighted residues indicate sites of incorporation of $^{13}C_5$-methionine.

sample	diffusion constant (m^2/s)	hydrodynamic radius (nm)	FA-TEG aggregation number
0.25 mM FA-TEG in D2O	(2.20 ± 0.02) x 10^-10	0.89 ± 0.02	1.8
1 mM FA-TEG in D2O	(1.35 ± 0.02) x 10^-10	1.45 ± 0.02	7.7
10 mM FA-TEG in D2O	(0.86 ± 0.01) x 10^-10	2.28 ± 0.03	30.3
74 µM CB2 in FA-TEG/lipid/CHS	(0.40 ± 0.03) x 10^-10	4.90 ± 0.06	
CB2 sample, second component	(0.03 ± 0.005) x 10^-10	65.4 0.89 ± 1.0	

Supplementary Table 1. Diffusion constants and derived hydrodynamic radii of Façade-TEG particles. Experiments were conducted at 22 ºC. The diffusion constants and derived hydrodynamic radii of FA-TEG particles indicate the critical micelle concentration of FA-TEG below 1 mM and an aggregation number in micelles at 10 mM FA-TEG of about 30. For the CB_2 sample, protein diffusion/hydrodynamic radie of micelles containing CB_2 is reported. The sample contained a second component with particles one order of magnitude larger in size.
